# A systematic review of electronic audit and feedback: intervention effectiveness and use of behaviour change theory

**DOI:** 10.1186/s13012-017-0590-z

**Published:** 2017-05-12

**Authors:** Timothy Tuti, Jacinta Nzinga, Martin Njoroge, Benjamin Brown, Niels Peek, Mike English, Chris Paton, Sabine N van der Veer

**Affiliations:** 1 KEMRI–Wellcome Trust Research Programme, Nairobi, Kenya; 2 Nuffield Department of Medicine, Oxford University, Oxford, UK; 3 Centre for Health Informatics, Division of Informatics, Imaging and Data Sciences, Faculty of Biology, Medicine and Health, Manchester Academic Health Science Centre, The University of Manchester, Manchester, UK; 4 MRC Health e-Research Centre, Farr Institute for Health Informatics Research, Manchester, UK; 5 NIHR Greater Manchester Primary Care Patient Safety Translational Research Centre, Manchester, UK

**Keywords:** Theory, Behaviour and behaviour mechanisms, Meta-analysis, Medical audit, Feedback, Performance, User-computer interface

## Abstract

**Background:**

Audit and feedback is a common intervention for supporting clinical behaviour change. Increasingly, health data are available in electronic format. Yet, little is known regarding if and how electronic audit and feedback (e-A&F) improves quality of care in practice.

**Objective:**

The study aimed to assess the effectiveness of e-A&F interventions in a primary care and hospital context and to identify theoretical mechanisms of behaviour change underlying these interventions.

**Methods:**

In August 2016, we searched five electronic databases, including MEDLINE and EMBASE via Ovid, and the Cochrane Central Register of Controlled Trials for published randomised controlled trials. We included studies that evaluated e-A&F interventions, defined as a summary of clinical performance delivered through an interactive computer interface to healthcare providers. Data on feedback characteristics, underlying theoretical domains, effect size and risk of bias were extracted by two independent review authors, who determined the domains within the Theoretical Domains Framework (TDF). We performed a meta-analysis of e-A&F effectiveness, and a narrative analysis of the nature and patterns of TDF domains and potential links with the intervention effect.

**Results:**

We included seven studies comprising of 81,700 patients being cared for by 329 healthcare professionals/primary care facilities. Given the extremely high heterogeneity of the e-A&F interventions and five studies having a medium or high risk of bias, the average effect was deemed unreliable. Only two studies explicitly used theory to guide intervention design. The most frequent theoretical domains targeted by the e-A&F interventions included ‘knowledge’, ‘social influences’, ‘goals’ and ‘behaviour regulation‘, with each intervention targeting a combination of at least three. None of the interventions addressed the domains ‘social/professional role and identity’ or ‘emotion’. Analyses identified the number of different domains coded in control arm to have the biggest role in heterogeneity in e-A&F effect size.

**Conclusions:**

Given the high heterogeneity of identified studies, the effects of e-A&F were found to be highly variable. Additionally, e-A&F interventions tend to implicitly target only a fraction of known theoretical domains, even after omitting domains presumed not to be linked to e-A&F. Also, little evaluation of comparative effectiveness across trial arms was conducted. Future research should seek to further unpack the theoretical domains essential for effective e-A&F in order to better support strategic individual and team goals.

**Electronic supplementary material:**

The online version of this article (doi:10.1186/s13012-017-0590-z) contains supplementary material, which is available to authorized users.

## Background

### Electronic audit and feedback

Audit and feedback (A&F) defined as the provision of clinical performance summaries to healthcare providers and organisations [1] is a well-used approach to support clinical behaviour change [2]. The increasing availability of health data in an electronic format (e.g. in Electronic Health Records), significantly increases potential for use of these data to provide electronic A&F (e-A&F).

e-A&F can be defined as the utilisation of interactive computer interfaces to provide clinical performance summaries to healthcare professionals [1, 3–7]. It aims to support the decision-making process or guide team management [3–7]. Although A&F is generally used when the patient is not present (e.g. like in bedside consultations, thereby making it distinctly different from computerized clinical decision support tools), e-A&F interventions specifically target clinicians or their managers and can aid improvement of patient care by providing timely or even real-time information for decision-making as part of operational management [8]. Furthermore, the interactive computer interface may allow users to filter, drill down and further explore their performance summaries.

Mechanisms of how A&F leads to behaviour change are variable and largely ignored in both individual and team-based contexts [9, 10]. While individual-based feedback is desirable [11], feedback to providers organised in teams or organisational units (e.g. whole facilities or departments) may offer a more scalable implementation model appropriate for low- and middle-income contexts [12]. In team-based care, multiple healthcare professionals are responsible for the same patients, and complex coordination is required [13]. Given previous A&F research showing team processes to explain more variance in outcome than practice structure[14], e-A&F interventions might additionally better facilitate improvement in team-based settings by addressing the aforementioned features.

### Use of theory

A&F is posited to increase accountability and quality of care through implicit behaviour regulation of healthcare professionals [9]—given it involves techniques of goal setting, monitoring and providing feedback [15]—and is postulated to be most effective when its design is guided by theory [9, 16, 17]. However, explicit use of theory in A&F interventions is scarce [18]. As a consequence, little is known on the more specific topic of how e-A&F interventions may enhance the quality of care.

It is noteworthy that barriers to behaviour change can be influenced by A&F [19] and that these barriers differ across clinicians, originating from differences in clinicians’ training, knowledge, work experience, personality and other individual characteristics. These barriers are complex and dynamic (they are influenced by ongoing changes in the healthcare organization which in turn influence clinicians’ behaviours) [20]. Use of theory can help direct predictions on the effect size of audit and feedback used to help clinicians’ behaviour change.

A&F interventions with graphical or written presentations, to our knowledge, provide feedback in the same format for all recipients. In this way, A&F is not sensitive to individual differences in barriers to behaviour change given the media platform. e-A&F could help address this individual-indifferent approach in applying theory to overcome this significant limitation for traditional A&F presentations [21].

However, when explicit theory underlying implementation interventions is absent, it may be possible to retrospectively identify the theoretical domains they were likely to target [22]. This can be achieved through use of broad theoretical frameworks, such as the Theoretical Domains Framework (TDF) [22, 23]. The TDF comprises 12 theoretical domains and 128 constructs from 33 behaviour change theories. It was developed using an expert consensus and validation process to identify an agreed set of theoretical domains that could be used in developing implementation interventions [22, 23].

We expect knowledge, skills, social/professional role and identity, beliefs about capabilities, environmental context and resources, beliefs about consequences, motivation and goals, behavioural regulation and nature of the behaviours and social influences TDF domains to be inherently targeted by e-A&F interventions. This expectation is informed by component theories such as normalisation process theory [24, 25] theory of planned behaviour [26] and control theory [27]. Our detailed justification for the selection of these domains is provided in Additional file 1. However, we are yet to come upon literature detailing how emotion domain was targeted by electronic quality improvement initiatives. Based on the context, not all domains might be relevant in all e-A&F interventions.

Identifying and summarising the theoretical concepts targeted by e-A&F interventions for primary and hospital-based care and exploring how these factors might influence the interventions’ effectiveness could contribute to better e-A&F design. Ultimately, this may lead to e-A&F to become a more reliable approach to improving the quality of clinical practice.

### Aim and objectives

We aimed to conduct a systematic review and meta-analysis of randomised controlled trials that evaluated the effectiveness of e-A&F interventions for clinical practice in primary care and hospital settings. Our objectives were to (1) assess the effect of these intervention on quality of care; (2) identify common aspects of the TDF employed as mechanisms of behaviour change in these intervention, and (3) explore links between identified TDF aspects, their nature or pattern of use across interventions and the magnitude of their effect size.

## Methods

We followed the Preferred Reporting Items for Systematic Reviews and Meta-Analyses (PRISMA) [28] statement for reporting our systematic review. PRISMA gives an evidence-based minimum set of recommendations for the reporting of systematic reviews and meta-analyses evaluating randomised trials, and can also be used as a basis for reporting systematic reviews of other types of research, e.g. evaluations of interventions [28].

### Criteria for considering studies for this review

#### Types of studies

Studies that assessed audit and feedback using randomised controlled trials (RCTs) were eligible for inclusion.

#### Types of participants

Studies involving feedback recipients who were healthcare professionals responsible for patient care were eligible for inclusion.

#### Types of intervention

Provider-oriented e-A&F interventions, defined as A&F interventions that utilised computer interfaces to provide clinical performance summaries to healthcare professionals, that specifically targeted behaviour change as part of clinical practice improvement were considered.

#### Types of outcome measures

Processes of care:

1. Dichotomous process measures. Percentage of patients receiving a target process of care (e.g. prescription of a specific medication, documentation of performance of a specific clinical task) or whose care was in compliance with overall clinical guidelines.
2. Continuous process measures. Any continuous measure of how providers delivered care (e.g. duration of antibiotic therapy, time to respond to a critical lab value).
  Outcomes of care
3. Dichotomous clinical outcomes. True clinical endpoints (e.g. mortality and development of a pulmonary embolism), as well as proxy endpoints, e.g. achievement of a target blood pressure or blood glucose level.
4. Continuous clinical outcomes. Various markers of disease or health status (e.g. mean blood pressure or cholesterol level).

#### Exclusion criteria

Studies whose focus was solely on non-clinical indicators (e.g. indicators on costs, financing, workload, coverage and time management), patient-reported experience measures, those that did not include an e-A&F arm component in case of a multi-faceted intervention and those that only reported feedback to patients were excluded. Non-electronic A&F, e.g. those delivering feedback verbally, by paper, telephone calls and electronic non-interactive A&F (i.e. they do not offer a computer interface which allows users to filter, drill down and further explore their performance summaries), e.g. emailed feedback were also excluded, as were studies that were not peer-reviewed or published in English.

### Data collection and analysis

#### Data sources and search strategy

We identified all relevant studies through a two-step search approach. An initial search strategy was developed based on MEDLINE indexed, informed by and including studies from the most recent Cochrane systematic review on A&F [2]. It was translated into the other databases using the appropriate controlled vocabulary as applicable (see Additional file 2 for the complete search strings and results). Reference lists of all included studies were also reviewed. We searched the following databases:

- MEDLINE and Ovid (1946 to August week 3 2016)—searched 12 August 2016
- EMBASE and Ovid (1974 to 2016 week 35)—searched 12 August 2016
- Cochrane Central Register of Controlled Trials (CENTRAL) 2016, Issue 8, part of The Cochrane Library. http://www.thecochranelibrary.com/, including the Cochrane Effective Practice and Organisation of Care (EPOC) Group Specialised Register—searched 12 August 2016
- CINAHL and EBSCOhost (1981 to present) searched 12 August 2016
- Science Citation Index and Social Sciences Citation Index, ISI Web of Science (1975 to present)—searched 12 August 2016

Search terms for *electronic* aspect of A&F were identified from running Ivers et al*.* search string [2], identifying Medical Subject Heading (MeSH) and common free-text terms used in studies with e-A&F. Through an iterative process, additional search terms from studies meeting our inclusion criteria were identified and used to strengthen the electronic filter.

#### Selection of studies

Two authors (TT and SV) independently screened the titles and abstracts against the inclusion criteria to identify potentially relevant studies. Where there was uncertainty, complete manuscripts were sought and disagreements were resolved through discussion. Full manuscripts underwent the same screening process by the same authors (TT and SV).

#### Data extraction and management

Data were extracted using a tailored version of EPOC’s data abstraction tool [29] by one reviewer (TT) and were checked by a second reviewer (MN); disagreements were resolved by discussion. Data extraction was guided by the EPOC data collection checklist [29], which we complemented with modifiable design elements of e-A&F suggested in previous systematic reviews [2, 28, 30]. We extracted data on: study design; study participants (e.g. cadre, team setup and clinical context); feedback characteristics (e.g. frequency of updates, interactive elements of the intervention, feedback content and reported benchmarks); intervention goals (baseline comparisons, direction of change, explicit action goal, etc.); reported effect size of primary outcomes only.

Two reviewers (TT and MN) independently assessed the risk of bias using Cochrane’s Review Manager software (V5.3) [31]. This included risk of selection bias (random sequence generation, allocation concealment and selection of two groups), reporting bias (blinding) and confounding (baseline characteristics and interventions). For each criterion, the study was classified as high risk of bias, low risk of bias or unclear risk of bias. An overall assessment of the risk of bias (low, medium and high risk of bias) was assigned to each of the included studies using the approach suggested in the *Cochrane Handbook for Systematic Reviews of Interventions* [32]. Studies with low risk of bias for all key domains or where at least four of the six criteria had low risk of bias with the other two not being attrition or reporting bias were considered to have a low risk of bias. Studies where risk of bias in at least one domain was unclear and at most three domains had low risk of bias were considered to have an unclear risk of bias. Studies with a medium risk of bias had three domains with low risk of bias that did not include attrition or reporting bias. Studies with a high risk of bias in at least four domains or random sequence generation bias, which decreased the certainty of the conclusions were considered to have a high risk of bias.

#### Identifying TDF domains

Two reviewers (TT, JN—a social scientist) independently extracted verbatim statements from the papers that referred to TDF domains that appeared to be targeted in the intervention and control arms, either explicitly or implicitly. These verbatim statements were summarised into TDF domains based on reported intervention elements and characteristics. The coding into domains was supported by evidence from the text, and inferences were made about which domains the authors intended to target in case this was not stated explicitly in the text. This was achieved by studying the descriptions of the interventions; each aspect judged to be targeting a domain with respect to the behaviours of clinician was coded (e.g. if social comparisons were used within e-A&F to evaluate clinician’s attitudes, abilities or performance relative to others, TDF’s domain ‘social influences*’* was inferred to have been target etc.). The 12 theoretical domains from TDF [23] informed coding and selection of relevant domains (see Additional file 3). Discrepancies in statement extraction and coding were resolved by discussion. For one study, a third reviewer (BB) independently extracted statements and coded them to verify the sturdiness of the coding process.

#### Data synthesis

Using the identified TDF domains, we analysed the commonly targeted aspects of TDF by looking at the frequency with which domains had been targeted in the studies. We also explored the nature and pattern of TDF domain use across the different studies and the associated magnitude of effect size. The reference table with the TDF domains and explanations that guided coding decisions is provided in Additional file 3. We descriptively reported the TDF aspects and primary outcomes’ effect sizes at the study-level, counting the number of times a domain had been identified across studies, and a descriptive analysis of potential links. We reported odds ratio reflecting adherence to desired practice from the primary dichotomous study outcomes.

For the quantitative meta-analysis, we assessed heterogeneity across studies to determine whether pooling of effect sizes was possible. Across studies, the effect size was weighted by the number of health professionals involved in the study reported to ensure that small studies did not contribute the same to the overall estimate as larger studies. Where the number of health professionals was not reported, the number of practices/hospitals was used instead. The summary statistics in the meta-analyses are reported as weighted odds ratio or weighted change relative to baseline control, weighted by the number of health professionals. This was supplemented by random effects univariate linear regression analysis used to explore potential sources of heterogeneity (e.g. intervention duration, feedback recipients, feedback frequency, feedback formats and theoretical domains targeted).

## Results

**Fig. 1:**
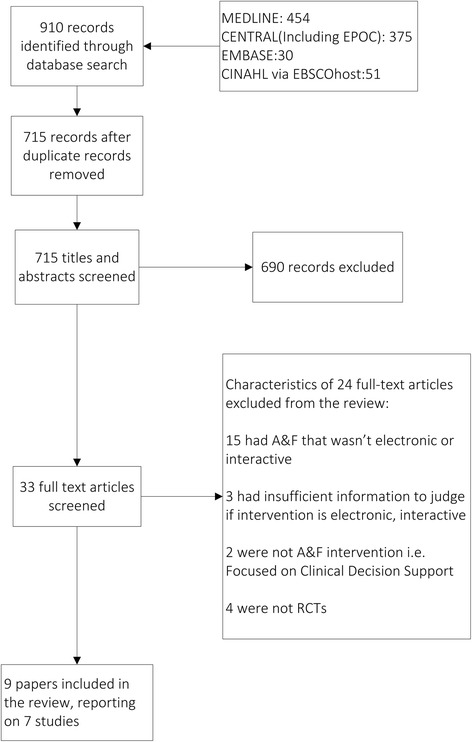
Flow diagram detailing process of including studies into the review

Our electronic searches yielded 715 unique papers, of which 33 were screened based on full text. Twenty-four papers were excluded after full review, and we included nine publications reporting the findings of seven studies (see Fig. 1).

### Description of studies and e-A&F interventions

Table 1 describes the characteristics of included studies and the e-A&F interventions they evaluated. Study settings varied, but all were from developed countries, with three studies conducted in very specialised settings, i.e. ancillary [33] and specialised cardiovascular units [34, 35] respectively. Only three out of the seven studies targeted interdisciplinary clinical teams [34–36].

**Table 1:** Characteristics of electronic audit and feedback interventions identified by the review

Study ID	Linder et al. 2010 [38]	Peiris et al. 2015 [40, 41]	Thomas et al. 2007 [39]	Carney et al. 2011 [33, 37]	Carlhed et al. 2006 [34]	Guldberg et al. 2011 [36]	Gude et al. 2016 [35]
Study design	CRCT	CRCT	CRCT	RCT	RCT	CRCT	CRCT
A&F domain	Prescribing and management of drugs, acute care management	Prescribing and management of drugs, guide primary prevention and screening	Chronic disease management	Guide primary prevention and screening	Acute care management	Chronic disease management	Chronic care management
Feedback recipients	Physicians	Physicians	Physicians	Physicians	Interdisciplinary clinical teams	Interdisciplinary clinical teams	Interdisciplinary clinical teams
Direction of change required	Reduce current behaviour	Increase current behaviour	Unclear	Reduce current behaviour	Increase current behaviour	Increase current behaviour	Increase current behaviour
A&F implementation	EHR-integrated tools	Web panel screens	Software panel screens	Web panel screens	Web panel screens	Distributed as software update	Web panel screens
Interactive A&F component	Drill down to patient level; select indicators for feedback	Drill down to patient level; select indicators for feedback	Choose presentation mode	Select indicators for feedback	Select indicators for feedback	Drill down to patient level; select indicators for feedback	Select indicators for action plan
Feedback presentation mode	Graphical summaries	Graphical summaries	Text summaries	Unclear	Graphical summaries	Tabular data, graphical summaries	List summaries, with ‘traffic light’ icons
Frequency of feedback updates	Monthly (automatically)	Bi-monthly (automatically)	Bi-monthly (automatically)	Every login	Real-time	3 times (month 1, month 3, month 12) during the trial by software update	Quarterly
Target goals basis	National CDC recommendations	Australian medical guideline recommendations	National evidence-based guideline recommendations	Radiologist defined their own goals	National evidence-based guideline recommendations	National evidence-based guideline recommendations	National evidence-based guideline recommendations
Action planning used	No	No	No	Yes	Yes	No	Yes
Benchmarks and comparators	Clinician vs peer average performance vs national benchmark (identified through expert analysis)	Peer-ranked performance data benchmarked against participating trial sites	Clinician vs aggregate average resident performance (based on Institute for clinical systems improvement targets)	Radiologist performance vs trial peers’ performance vs achievable national benchmarks	Local team vs average trial peers’ performance; local team vs national average performance	Local practice performance vs average performance of trial peers	Local team-based performance vs. peer performance (calculated as achievable benchmarks)
Intervention duration (months)	9	12	12	21	18	14	19.8 and 22.5^a^
Participants in study (*n*)	Primary care practices (27)	Primary healthcare centres (60)	Clinical groups (residents) within hospital	Clinical groups (radiologists) between regional hospitals	Multi-disciplinary teams between hospitals (38)	General practices (86)	Cardiac rehabilitation Centres (18)
Outcome type	Dichotomous process measures	Dichotomous process measures	Dichotomous process measures	Dichotomous process measures	Dichotomous process measures	Dichotomous process measures	Dichotomous process measures and patient outcomes
Comparator arm	A&F vs current practice	A&F vs current practice	A&F vs current practice	A&F vs current practice	Head-to-head comparison of A&F	A&F vs current practice	Head-to-head comparison of A&F
Role of e-A&F in overall QI strategy^b^	Core	Moderate	Minimal	Core	Moderate	Core	Core
Baseline performance known	Unclear	Yes	Yes	Yes	Yes	Yes	Yes
Risk of bias	Medium	Low	Medium	Unclear	High	Medium	Low

Abbreviations: *A&F* audit and feedback, *CRCT* cluster randomised controlled trial, *RCT* randomised controlled trial, *CDC* Centre for Disease Control, *QI* quality improvement

^a^Average length of study period per centre in both arms of the intervention (from table 2 [35]). ^b^On a 3-point scale (minimal, moderate and core).

Study-specific risk of bias assessment can be found in Additional file 4

Benchmarks provided in the e-A&F reports most commonly offered comparisons of individual performance versus average local and national performance [33, 34, 37–39], or local site performance versus performance of all participating study sites [36, 40, 41]. Our definition of benchmarks is defined in detail elsewhere [42]. If there were other quality improvement (QI) strategies used, we assessed the extent to which one would reasonably consider e-A&F to be the key intervention to which the effect size would be attributed. Three categories identified were: (1) whether e-A&F was optional (minimal), (2) whether e-A&F was mandatory but included other QI interventions most of which were not implemented within e-A&F (moderate), (3) whether e-A&F was mandatory and included other QI interventions most of which were implemented within e-A&F (core).

In two studies, the intervention allowed clinicians to set their own goals or actions and track them [34, 35], with the rest utilising guidelines from professional bodies and/or evidence from previous studies as the study goals.

With regard to their interactive characteristics, six e-A&F interventions allowed recipients to select which additional indicators to include in their feedback report, in three cases feedback recipients, could drill down to specific patient population details [36, 38, 40, 41]. The implementation of the e-A&F interventions varied in design and form. Four studies created web panels containing patient data to be used for A&F, with one using stand-alone software program [39], and another one implementing an integrated EHR tool [38]. One study combined the electronic performance data with a software program that rendered it digitally and distributed these ‘updates’ regularly [36]. Interventions that had not been integrated in electronic health records had a separate process of data collation, with data being queried from medical records to populate a separate e-A&F tool.

**Fig. 2:**
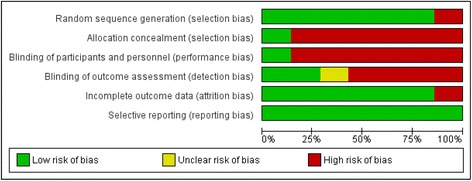
Risk of bias graph. Review authors’ judgements about each risk of bias item presented as percentages across the seven included studies. Study specific bias assessment can be found in Additional file 3

In describing the control arm of the study, only three studies went beyond stating usual care and gave a clear detailed description of what the intervention was being tested against [34, 35, 39]. None of the studies randomised feedback design elements within intervention arms, but one did compare outcomes within the intervention arm based on rate of use of e-A&F [38]. Of the seven studies included, studies with the highest number of participants [35, 41] had a low risk of bias; three had a high risk [34, 36, 38] (see Fig. 2). The most common sources of a high risk of bias related to blinding of participant and personnel selection bias; clarity of reporting regarding the risk of bias was often insufficient (see Additional file 4 for a summary of risk of bias assessments across studies).

### Effect of e-A&F interventions on quality of care

Table 2 displays the effect size and associated 95% confidence interval (95% CI) for each included study. Three studies found a positive effect of the e-A&F intervention on the quality of care. Peiris et al. reported a statistically significant difference of 9.4% (OR 1.47, 95% CI 1.41–1.53) between the intervention and control group for the number of patients who received appropriate screening of cardiovascular risk [40, 41]. Thomas et al. reported a statistically significant difference of 13% (OR 1.72, 95% CI 1.20–2.47) and 11.7% (OR 1.75, 95% CI 1.18–2.59) between the intervention and control group for patients who received appropriate haemoglobin and cholesterol testing, respectively [39]. Carlhed et al. reported statistically significant difference of 10.6–14.9% between the intervention and control group in four out of five Swedish national guideline-derived quality indicators of acute myocardial infarction [34]. None of the other studies found an effect of the intervention on *all* the outcome measures evaluated.

**Table 2:** Primary outcomes of the identified studies and the reported effect size

Study ID	Intervention sample size; control sample size	Outcome of interest	Odds ratio (95% CI)
Linder et al. 2010 [38]	I: 258 clinicians; C: 315 clinicians	(1) Antibiotic prescribing rate for acute respiratory infection	0.97 (0.92–1.03)
Peiris et al. 2015 [41]	I: 19385 patients; C: 19340 patients	(1) Patients who received appropriate screening of cardiovascular risk factors by the end of study	1.47 (1.41–1.53)
	I: 5335 patients; C: 4846 patients	(2) Prescription rate of recommended medications for high-risk cohort	1.25 (1.16–1.35)
Thomas et al. 2007 [39]	I: 252 patients; C: 231 patients	(1) Diabetes care metrics for all participating residents’ patients at study inception and completion including haemoglobin monitoring in the prior 6 months	1.72 (1.20–2.47)
		(2) Diabetes care metrics for all participating residents’ patients at study inception and completion lipid monitoring in the prior 12 months	1.75(1.18–2.59)
Carlhed et al. 2006 [34]	I: 3786 patients; C: 2940 patients	(1) Lipid-lowering therapy at discharge	3.26 (2.49–4.27)
		(2) Angiotensin-converting enzyme (ACE) inhibitors at discharge	10.08 (7.31–13.90)
		(3) Clopidogrel at discharge	1.96 (1.77–2.18)
		(4) Heparin or low-molecular weight heparin (LMWH) during hospitalisation	3.47 (2.89–4.16)
		(5) Performed coronary angiography (or, for hospitals lacking in-house coronary angiography, referral to another hospital)	3.05 (2.57–3.63)
Guldberg et al. 2011 [36]	I: 1196 patients; C: 1050 patients	(1) Haemoglobin measurement sustained	0.86 (0.59–1.25)
	I: 121 patients; C: 91 patients	(2) Haemoglobin measurement initiated if no measurement at baseline	0.77 (0.45–1.33)
	I: 1109 patients; C: 887 patients	(3) Cholesterol measurement sustained	1.74 (1.35–2.24)
	I: 208 patients; C: 258 patients	(4) Cholesterol measurement initiated if no measurement at baseline	2.07 (1.38–3.12)
Carney et al. 2011 [33]	I: 23 radiologists: C: 9 radiologists	(1) Mean recall rates at time T1(0–9 months)	1.12 (1.00–1.27)
		(2) Mean recall rates at time T2(9–18 months)	1.10 (0.96–1.25)
Gude et al. 2016 [35]	I: 7341 patients; C: 4591 patients	(1) Complete data on psychological functioning	1.07 (0.46–2.5)
	I: 7341 patients; C: 4591 patients	(2) Complete data on social functioning	7.95 (0.54–116.3)
	I: 7341 patients; C: 4591 patients	(3) Complete data on lifestyle factors	1.11 (0.45–2.75)
	I: 4934 patients; C: 4071 patients	(4) Disease specific education completed^a^	0.57 (0.31–1.06)
	I: 5580 patients; C: 4591 patients	(5) Lifestyle modification programme completed^a^	1 (0.48–2.04)
	I: 4591 patients; C: 7341 patients	(6) Improved quality of live after CR	0.99 (0.84–1.19)
	I: 7341 patients; C: 4591 patients	(7) Successful smoking cessation	1.02 (0.86–1.2)
	I: 7341 patients; C: 4591 patients	(8) Patients receive a discharge letter with remaining lifestyle goals	0.87 (0.27–2.81)
	I: 4591 patients; C: 7341 patients	(9) Complete data on physical functioning	1.32 (0.45–3.84)
	I: 4591 patients; C: 7341 patients	(10) Complete data concerning cardiovascular risk factors	1.2 (0.65–2.23)
	I: 2922 patients; C: 7341 patients	(11) Exercise training completed^a^	1.64 (0.57–4.71)
	I: 4071 patients; C: 4898 patients	(12) Relaxation and stress management training completed^a^	0.44 (0.14–1.41)
	I: 4591 patients; C: 7341 patients	(13) Cardiovascular risk factors evaluated at discharge	1.22 (0.4–3.76)
	I: 4591 patients; C: 7341 patients	(14) Improvement in exercise capacity	0.86 (0.69–1.07)
	I: 4591 patients; C: 7341 patients	(15) Successful work resumption	1.04 (0.86–1.24)
	I: 4591 patients; C: 7341 patients	(16) Moderately active lifestyle norm met at discharge	1.03 (0.82–1.29)
	I: 4591 patients; C: 7341 patients	(17) Vigorously active lifestyle norm met at discharge	0.88 (0.74–1.04)

Note: *I* intervention arm, *C* control arm. ^a^Excluded centres with incomplete data for this outcome

**Fig. 3:**
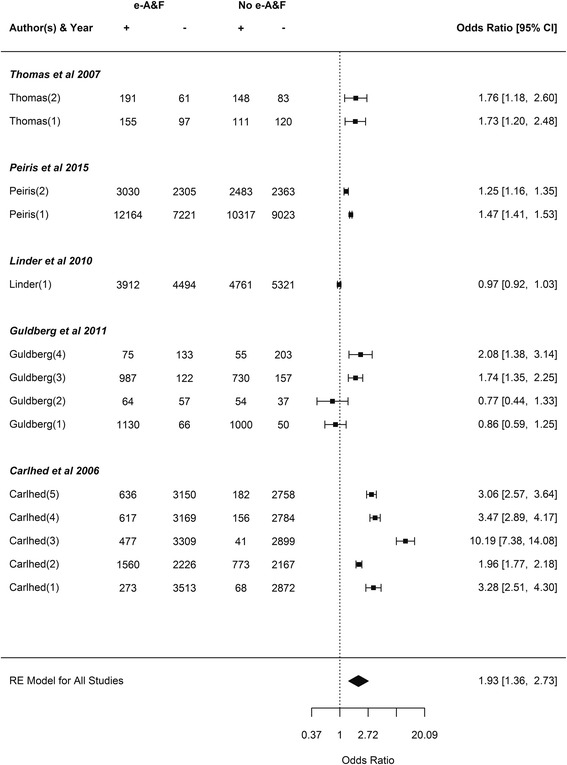
Forest plot of e-A&F targeting quality improvement of team practice. Descriptions of outcomes as annotated in the brackets can be found in Table 2. Due to the high variation as illustrated by *I* ^2^ value, the average effect should not be considered reliable

The weighted odds ratios of each primary outcome for each study and all studies combined for e-A&F are shown in Fig. 3, with substantial heterogeneity observed across studies (*P*_heterogeneity_ < 0.001, *I* ^*2*^ = 99.12%, 95% CI: 98.25–99.68). The weighted odds ratio of compliance with desired practice was 1.93 (95% CI: 1.36–2.73) when considering e-A&F to no A&F. Please note that due to the high variation as illustrated by *I* ^2^ value, this average effect should not be considered reliable. We could not reduce the heterogeneity by considering subsets of outcome measures. The summary odds ratio of e-A&F comparing the intervention arm with access to e-A&F to control arm without the same access was highly unreliable due to high heterogeneity. Carney et al. was omitted from meta-analysis due to missing information in their report, i.e. they did not include the number of screening mammograms with a recommendation for immediate follow-up (a positive result) for both intervention and control arms of the study at intervals 1 and 2 [33, 37]. In Gude et al. [35], both arms of the study received e-A&F (but for different sets of outcomes, and so were each other’s control). Given the evidence of contamination effect that A&F might have on overall quality of care in general even if a subset of indicators are being tracked (Susan Gachau, et al., Effects of audit and feedback delivered within an emerging clinical network in Kenya on multiple indicators of the process of paediatric hospital care – a longitudinal observational study. BMJ Quality and Safety, submitted), and the admission in this study’s report of risk of contamination, it was omitted from the meta-analysis.

Further exploration of possible sources of heterogeneity as detailed in previous reviews [2] showed the number of theoretical domains targeted in the control arm, feedback characteristics (graphical feedback, A&F head-to-head comparison and real-time feedback frequency) and intervention duration to be the biggest explicators of the level of heterogeneity (Table 3). The components of the meta-regression reported in Table 3 were tested univariately, i.e. each separately from one another. A multivariable meta-regression model adjusting for effects of all components on intervention effect was not possible due to the small number of studies (*n* = 5) and outcomes (*n* = 14) included in the meta-analysis.

**Table 3:** Random effects meta-regression model

Covariate	*I* ^2^ residual heterogeneity/unaccounted variability	*R* ^2^ amount of heterogeneity accounted for	*P* value (moderator)
Null model	99.12%	–	–
Size of clinical teams	98.97%	**0.00%**	0.884
Intervention duration	98.03%	51.70%	0.001**
Interdisciplinary teams	98.96%	**10.40%**	0.142
Real-time feedback^a^	97.81%	58.04%	0.001**
Graphical feedback	99.24%	**0.00%**	0.825
TDF			
No. of intervention domains coded	98.79%	**0.00%**	0.723
No. of control domains coded	97.65%	60.81%	0.001**

Note: Model is a univariate regression. ^a^Result is also the same for local goals and ‘A&F head-to-head comparison.’ ‘Significance codes: 0 ‘***’, 0.001 ‘**’, 0.01 ‘*’, 0.05’

### Common aspects of TDF employed as change behaviour mechanisms in e-A&F

Only two studies explicitly reported using theory (adult learning theory and control theory) to inform intervention design and reported to have tested theoretical concepts with the trial [33, 35]. The coding for the domains targeted in the intervention and control groups for each of the studies is shown in Table 4 below. The reference table with the TDF domains and explanations that guided coding decisions is provided in Additional file 3.

**Table 4:** Theoretical domains constructs identified from studies included in analysis

Study	Intervention arm		Control arm	
	Domain	Support statement/action	Domain	Support statement/action
Linder et al. 2010 [38]	(1) Knowledge	(1) CDC^a^ recommendations (statements having factual or procedural knowledge)	Usual care	
	(4) Nature of the behaviours	(4) Targeted behaviour was reduction of inappropriate antibiotic prescribing;		
	(5) Beliefs about consequences;	(5) Included billing data to provide a sense of a financial incentive to clinicians; incorrect beliefs that antibiotics are necessary to treat acute respiratory infections		
	(6) Motivation and goals; (9) Social influences;	(6, 9) View displayed a clinician’s performance against his or her clinic peers and against national benchmarks;		
Peiris et al. 2015 [41]	(1) Knowledge	(1) Synthesis of recommendations from several screening and management guidelines for cardiovascular diseases, kidney disease and diabetes mellitus	Usual care	
	(2) Skills (8) Environmental context and resources;	(2, 8) Practices received an average of 48-min support per month comprising on-site training, remote clinical webinars and helpdesk access;		
	(6) Motivation and goals (9) Social influences	(6, 9) Health services could view peer-ranked performance data benchmarked against other participating trial sites;		
	(7) Memory, attention and decision process; (11) Behaviour regulation	(7, 11) Tool to allow health services to audit health records, identify performance gaps rapidly and establish recall/reminder prompts rapidly. Provided point of care recommendations based on cardiovascular diseases risk (11) Identification of screening and management gaps for the whole patient population built into a commonly used audit tool		
	(12) Nature of behaviours	(12) Shifting prescribing behaviours		
Thomas et al. 2007 [39]	(1) Knowledge (2) Skills	(1, 2) Dashboard information was organised by evidence-based guidelines, highlighting relevant data; received usual clinic education consisting of faculty review of diabetes care among patients supervised with the resident. (1, 2) Two 1-h sessions introducing registries, describing their value in practice improvement, and providing instruction on registry use	(1) Knowledge; (2) Skills (8) Environmental context and resources	(1, 2, 8) Received usual clinic education consisting of faculty review of diabetes care among patients supervised with the resident and linked to access to the ‘electronic curriculum for diabetes care’;
	(6) Motivation and goals; (9) Social influence;	(6, 9) Feedback comparing their diabetes performance metrics to aggregate resident performance;		
	(8) Environmental context and resources;	(8) Access to the ‘electronic curriculum for diabetes care’ linked to electronic registry feedback;		
	(11) Behaviour regulation	(11) Registry-generated lists identifying patients not in compliance with guideline recommendations		
Carney et al. 2011 [33]	(1) Knowledge; (2) Skills; (8) Environmental context and resources;	(1,2,8) Continuous medical education modules	Usual care	
	(5) Beliefs about consequences	(5) Profiled breast cancer risk in each radiologist’s respective patient population; Information on the possible impact of medical malpractice concerns on recall rates		
	(6) Motivation and goals	(6) Awarded 2 h of category I continuous medical education credit;		
	(7) Memory, attention and decision process	(6, 7) Radiologists were able to insert their goals for changes;		
	(9) Social influences	(6, 9) Audit data individualised for each participating radiologist with comparisons to both national benchmarks and to peers for the same measures during the same time period.		
	(11) Behaviour regulation	(11) Illustrating the metrics in clinical performance that could be improved; reinforce change by assisting radiologists to develop goals that would improve their performance;		
	(12) Nature of behaviours	(12) Reduce unnecessary recall from memory practice		
Carlhed et al. 2006 [34]	(1) Knowledge	(1) Educational on the content of National Acute Myocardial Infarction guidelines	(1) Knowledge	(1) Educational on the content of National Acute Myocardial Infarction guidelines
	(4) Belief about capabilities (6) Motivation and goals	(4, 6, 9) During and between learning sessions, the teams were requested to come up with action plans for appropriate local changes; (6, 9) Local performance feedback with comparisons to other centres and national average	(4) Belief about capabilities (6) Motivation and goals	(4, 6, 9) During and between learning sessions, the teams were requested to come up with action plans for appropriate local changes;
	(8) Environmental context and resources	(8) Education training partly managed at a web-based portal and linked with the registry web tool.	(9) Social influences (11) Behaviour regulation	(9, 11) Frequent collaborative approach meetings to solve common problems between teams and results and lessons learnt were shared with other team members;
	(9) Social influences (11) Behaviour regulation	(9, 11) Frequent collaborative approach meetings to solve common problems between teams and results and lessons learnt were shared with other team members;		
Guldberg et al. 2011 [36]	(1) Knowledge	(1) Guidelines concerning treatment and control of type 2 diabetes in general practice		
	(5) Beliefs about consequences;	(5) Graphic treatment history of the individual patients by each variable		
	(6) Motivation and goals;	(6) To provide an overview of the patient population as a basis for planning interventions if needed		
	(7) Memory, attention and decision process	(7) Option to use data in patient consultations		
	(9) Social influences;	(6, 9) Graphic presentations comparing each clinic with the other participating clinics by each variable		
Gude et al. 2016 [35]	(1) Knowledge	(1) Ineffectiveness partly explained by the fact that professionals were not able to translate their intentions into completed actions, i.e. the second step of the mechanism, before the study end.	^a^ *Exact replica of the intervention arm*	
	(6) Motivation and goals (intention) (11) Behaviour Regulation	(6) The intervention successfully encouraged teams to define local performance improvement goals (6, 11) Educational outreach visits were held with the local multidisciplinary team to set goals and plan actions and update existing action plans		
	(7) Memory attention and decision processes	(7) Educational outreach visits were held with the local multidisciplinary team to reflect on the feedback. The team discussed and reflected upon their most recent feedback report and created or updated their QI plan.		
	(8) Environmental context and resources	(8) Some of the persisting organisational barriers were related to lack of resources (e.g. budget ceilings imposed by insurers), competing interests between managers from different clinical disciplines, and poor attendance of clinical leadership (cardiologists and managers) at outreach visits. (8) Implementation of web tool to be used to develop a quality improvement plan by selecting indicator areas for improvement (related to quality indicators in the feedback report)		
	(9) Social influences	(9, 6) Performance scores based on the centre’s performance score relative to peer performance was implemented using the concept of achievable benchmarks.		
	(12) Nature of behaviours	(12) Update existing action plans following a continuous audit and feedback improvement cycle		

Note: In the ‘support statement/action column’, the preceding number(s) in the brackets represent the numbered domain in the ‘domain’ column and reference number for domain explanations found in Additional file 2. ^a^ *CDC* Centre for Disease Control, USA. In Gude et al**.** [35], both arms received the e-A&F intervention, whilst serving as each other’s control

Table 5 presents the number of times each of the domains were coded for both arms in each included study. For five studies, we identified at least six domains identified in the intervention arm [33–35, 37, 39–41]. The study informed by adult learning theory had the most domains identified in the intervention arm, but did not describe its control arm with the same rigour [33].

**Table 5:** Identified pattern and frequency of theoretical domains

Study ID	Linder et al. 2010 [38]	Peiris et al. 2015 [40, 41]	Thomas et al. 2007 [39]		Carney et al. 2011 [33, 37]	Carlhed et al. 2006 [34]		Guldberg et al. 2011 [36]	Gude et al. 2016 [35]		Total
Domain	Intervention	Intervention	Intervention	Control	Intervention	Intervention	Control	Intervention	Intervention	Control	
(1) Knowledge	1	1	1	1	1	1	1	1	1	1	10
(2) Skills		1	1		1						3
(3) Social/professional role and identity											0
(4) Beliefs about capabilities						1	1				2
(5) Beliefs about consequences	1				1			1			3
(6) Motivation and goals	1	1	1		1	1	1	1	1	1	9
(7) Memory, attention and decision process		1			1			1	1	1	5
(8) Environmental context and resources		1	1	1	1	1			1	1	7
(9) Social influences	1	1	1		1	1	1	1	1	1	9
(10) Emotion											0
(11) Behaviour regulation		1	1		1	1	1		1	1	7
(12) Nature of the behaviours	1	1			1						3
Total	5	8	6	2	9	6	5	5	6	6	

Note: All control arms of studies that had not been described beyond ‘usual care’ ended up with 0 coded domains in control arm

The most frequently coded domains in the intervention arm were ‘knowledge’, ‘motivation and goals’ and ‘social influences’ (all seven studies). The knowledge domain was also coded for the two studies that included a description of the intervention in the control arm [34, 39]. The most commonly coded domains when intervention and control arm of trials were combined were knowledge (coded ten times) and motivation and goals and social influences (both coded nine times). We did not identify any studies whose interventions targeted ‘social/professional role and identity’ or ‘emotion’.

Of the three studies that found a positive effect of the e-A&F intervention on the quality of care, one had the second highest number of coded domains in intervention arm [40, 41], and the other two were the only studies with domains coded in both intervention and the control arm [34, 39]. The low number of studies identified did not allow any inferences about patterns of theoretical domains identified and their link with effect sizes.

## Discussion

### Summary of findings

Our meta-analysis of five studies revealed the included electronic audit and feedback (e-A&F) interventions to be highly heterogeneous, even when subsets of outcome measures were considered. The weighted pooled odds ratio of compliance with desired practice was 1.93 (95% CI 1.36–2.73) when considering e-A&F to no A&F. This pooled average effect suffered from distortion as studies had varied sizes, differed in results and tended to be biased. Additionally, the meta-regression results would likely be biased given that they tend to have poor performance where there are few studies [43]. We therefore considered this average effect to be unreliable. Using the theoretical domains framework (TDF) to identify the theoretical concepts underlying the interventions, we found that the TDF domains of knowledge, motivation and goals and ‘social influences’ were most commonly targeted; professional identity and emotion were not targeted by any of the interventions. Due to the small number of studies identified, inferences about patterns of domains and their link with effect sizes could not be made.

### Relation to other studies

To our knowledge, we are the first to perform a meta-analysis of the effectiveness of e-A&F interventions. Ivers et al. [2] identified 140 randomised controlled trials (RCTs) of A&F interventions that objectively measured provider performance in a healthcare setting. However, whereas they included feedback in any format, we focused on A&F interventions that were delivered electronically. Two studies included in our review [36, 39] were also identified by Ivers et al. Their meta-regression of studies featuring dichotomous outcomes included 82 comparisons from 49 studies. For dichotomous outcomes, Ivers et al. found the weighted median risk difference to be 4.3% increase in compliance with desired practice, unlike our results where we could not determine a reliable average effect due to high heterogeneity. This difference might partly be due to their exclusion of studies with a high risk of bias from analysis whereas we included all the studies with sufficient baseline information. Our exploration of sources of heterogeneity yielded similar findings to Ivers et al. on significance of instructions for improvement and feedback frequency for A&F. However, the inability to make firm conclusions from the analysis of heterogeneity due to the indirect nature of the comparisons is common in both studies. Our heterogeneity findings are further compounded by the small number of identified studies. We identified two more intervention characteristics (number of TDF elements on control arm and intervention duration) as being possible sources of heterogeneity in e-A&F interventions. The electronic and interactive component of feedback—which they did not evaluate—captures key aspects of feedback possibly associated with improved effectiveness. Specifically, e-A&F facilitates auto-delivery of feedback more frequently than other formats, including offering real-time updates; offers the ability to easily track measurable practice goals and adherence to specific action plans in real-time and is customisable. Additionally, e-A&F overcomes the pragmatic consideration of additional costs associated with providing personalised feedback more frequently, which plays into its added effectiveness.

Colquhoun et al. [15] examined the extent to which explicit theory was used in the 140 RCTs of A&F interventions identified in Ivers et al.’s review. In contrast to our study, they limited their approach to explicit use of theory and only included 14% of trials (*n* = 20), similar to what we found with only two out of seven studies explicitly stating that theory informed the design of their intervention [33, 35]. In contrast to Colquhoun et al*.*’s approach of classifying theories by application field, TDF represents common psychological aspects that most theories target. Our approach broadened the scope from explicit theory use while focusing on e-A&F. This was motivated by evidence showing e-A&F to influence contextual effect modifiers and intervention design [9, 21] but at the same time providing limited insight of how they can best be aligned to provide feedback supporting clinical practice, given their increasing popularity.

There are other examples of efforts to use TDF within systematic reviews such as Little et al. [22], which examined theoretical factors that post-fracture interventions aimed at patients at risk of osteoporosis, but did not include physician-directed A&F component. Similar to our study, they applied TDF retrospectively to explore implicit use of theoretical domains. They identified five commonly targeted domains out of the possible fourteen, with all studies targeting at least four out of the five domains identified. In line with Little et al., we found that all our studies targeted knowledge and social influences domains. While they found an inverse relationship for both number of times and number of different domains coded and the effect size, our analysis found the number of different domains coded *in the control arm* might be considerably associated with effect size, with the number of unique domains coded in intervention arm having no effect. However, this difference might be due to the risk of bias of studies included in review—although they did not report on risk of bias assessment. Also, the heterogeneous nature of studies in our review is possibly substantively higher than in Little’s review and might also account for the differences.

### Theoretical concepts targeted by electronic audit and feedback

Knowledge, motivation and goals and social influences were the most frequently coded domains. In most studies included in our analysis, national guidelines determined the desired state of practice, rather than localised action-planning and goal setting. This is consistent with other studies where investigators concluded that clinical teams lacked key knowledge about practice needed to improve behaviour [35]. At the same time, goal setting recommendations propose clinicians not to be highly motivated to achieve evidence-based population-level quality targets, but instead tend to prioritise competing organisational and clinical goals [44].

Our findings related to common theoretical domains may be indicative of the belief that inclusion of local and national peer performance comparators offered a sense of importance and urgency of outcomes for teams to work towards. This reflects how feedback linked to team roles is part of a broader transformation of any clinical team, and an acknowledgement of the behavioural unit the team represents [13]. Hysong et al. argued that there is a need to understand how changes in the individual’s performance impact team outcomes, and if and how feedback practices are aligned to support teams [13]. Additionally, the studies possibly assumed that using peer ranking as social comparisons of practice behaviour would (1) instil a conscious desire among team members to maintain a certain degree of similarity in performance; and (2) help highlight a distinctive pattern of culture and practice behaviour shared by team members. One study illustrated how performance closer to benchmarks motivated change in practice [33]. This is consistent with evaluations of team practice behaviour which show improved perceptions of effectiveness and appreciable changes in practice performance where clinical teams have been regarded as a behavioural unit rather than individuals [13, 34]. Gauging the level of interdependence, enabling efficient care coordination and encouraging parity among all individual clinicians in the quality improvement endeavour require insight into change mechanisms involved in setting a shared quality agenda [45]. However, our results do not highlight how peer ranking as a social pressure encouraged goal setting as part of regular behaviour for team members.

With regards to differences in use of theory across studies, implicit targeting of ‘memory’, attention and ‘decision process’ and ‘behaviour regulation’ domains represents particularly intriguing findings. Within the identified e-A&F studies targeting memory, attention and decision process, those with explicit use of theory in intervention design found no significant differences between the study arms compared to studies without explicit theory use which reported significant differences. Behavioural regulation domain, which is a fundamental pillar of how and why feedback purportedly works, could not be confidently identified from two studies [36, 38]. Where the processes of goal selection, prioritisation and monitoring, coupled with action planning were not included in the feedback process, it is difficult to ascertain the active components that had a significant effect on outcomes [9]. This might signal a tendency to overestimate the impact of theoretical domains on outcome effect where active components of A&F are not well defined or targeted [2, 9]. It can also be indicative of how lack of adoption of a menu of theoretical domains in intervention design limits the ability to validate each domain within the context of e-A&F [46]. However, due to the small number of studies identified, it was difficult to theorise the relationship between the differences in theoretical domains targeted across studies and their impact on the effect size.

Professional identity and emotion were rarely coded, although we presumed emotion domain would not feature in e-A&F interventions given the lack of evidence within digital health on how it has previously been targeted. This was indicative of how clinical team practice behaviours might have been assumed not to be influenced by these factors. Yet, these domains are posited to influence clinical practice [47–49]. Addressing this gap in future studies might further increase the understanding of effect of e-A&F interventions on practice.

### Implications for practice and future research

Feedback reports delivered electronically have the potential to deliver adaptive feedback to individual team members [13, 21]. The possibility for individual clinicians to track personal goals while still aiming to conform to group performance targets implicitly imposes expectations for future designs of e-A&F that: (1) these interventions offer the ability to capture the intentions of team members at an individual level, and (2), they might be more informative if they cater for the evaluation of individual-team goal setting interaction. As such, future studies on e-A&F should aim to conduct head-to-head comparisons between individual versus team spanning: (i) goal attainment–where the feedback recipient has individual targets apart from the team’s, (ii) differences in frequency of updating target goals and nature of goals pursued and (iii) differences in memory, attention and decision process as delivered by e-A&F and in light of contextual effect modifiers.

The rationale for the interventions in our study and in some cases, how interventions were delivered was sometimes inadequately described. Descriptions of the control group specifically were often absent. This persistent problem in lack of descriptive clarity in A&F studies [50] makes it difficult to disentangle the active ingredients of the interventions from the delivery method [9]. This curtails the ability to identify the true underlying nature of observed (lack of) behaviour changes, and it constrains the studies’ replication in wider settings [2, 51, 52]. Future studies should therefore employ explicit use of theory in designing and evaluating A&F interventions as a clear effort to improve upon understanding of A&F mechanisms of action [9].

Additionally, testing of various theoretical concepts in a multi-component e-A&F interventions is now feasible through approaches such as AB testing[51]. Future e-A&F studies ought to consider stepwise research designs, which embed tuple-wise testing of theoretical domains within audit cycles. This would allow determination of separable direct additive effects of each domain on practice behaviour. Also, varying frequency, content and delivery of feedback would help inform future intervention designs [9].

### Limitations

The search strategy used to identify studies included a newly developed filter for identifying electronic interventions. As there is no consensus in definitions and terms used to describe e-A&F, we cannot be certain that we did not miss studies based on the search terms we used. However, the rigour of the approach used for developing the electronic filter, coupled with an A&F filter which has been used in a Cochrane review strengthened our search strategy [2]. We manually screened all included A&F trials in Ivers et al’s review to ensure that the search had picked up all e-A&F studies.

Due to small numbers, we included five studies in the meta-analysis regardless of their risk of bias. As the one study with a low risk of bias was also the one with the highest weight in the analysis, we deemed a sensitivity analysis to be non-informative. However, we also examined whether differences in the level of the unit of analysis (groups of professionals/individual professionals versus patients) was a source of heterogeneity, since analyses conducted at different levels can result in different effect estimates. Overall, in hindsight, there is an argument for not doing a meta-analysis at all given the high levels of heterogeneity and the small number of studies identified. We cannot make a conclusion that electronic feedback is better than any other type of feedback, e.g. written or verbal.

## Conclusions

We conclude that although opportunities for electronic A&F are becoming more common, e-A&F randomised interventions are scarce, and from our findings, highly heterogeneous. e-A&F that have been implemented and tested in trials to support performance improvement of clinical practice tend to implicitly target only a fraction of known TDF concepts. This is further compounded by poor assessment of comparative effectiveness of targeted theoretical concepts across trial arms and high risk of bias of the studies.

This is in spite of common theoretical frameworks creating a basis for operationalization of computerised tailoring of A&F for practice behaviour change [21]. Future research should seek to unpack the distinctions between individual and team-based electronic A&F, including issues such as task ownership in addressing personal and team targets, behavioural distinctions in prioritising individual, team and national performance goals, and the influence of professional role, identity and intentions of team members on individual- and team-centric clinical performance goals. Research should also seek to utilise e-A&F capabilities for evaluating of various theoretical concepts in a multi-component interventions using approaches such as AB testing.

## Additional files

Additional file 1: Table S1. Component theories associated with audit and feedback [53–55]. (DOCX 19 kb)

Additional file 2: Table S2. Search strategy. (DOCX 62 kb)

Additional file 3: Table S3. TDF domains and constructs. (DOCX 55 kb)

Additional file 4: Study specific bias assessment. (TIF 5143 kb)
