# Supplementary material for: A systematic review of electronic audit and feedback: intervention effectiveness and use of behaviour change theory
Source: Implement Sci. 2017 May 12;12:61. doi: 10.1186/s13012-017-0590-z (PMC5427645; doi:10.1186/s13012-017-0590-z)
Supplement: Supplementary file 1 — Component theories associated with audit and feedback [53–55]. (DOCX 19 kb) [file 13012_2017_590_MOESM1_ESM.docx]

## **S1: Component theories associated with Audit and Feedback**

| **Supplementary Table 1: Component theories informing selection of theoretical domains posited to be associated with audit and feedback** | | |
| --- | --- | --- |
| **TDF Domain** | **Example theory informing this domain** | **Support for selecting this domain given example theory** |
| (1) Knowledge | Normalisation Process Theory | NPT Collective action's skill set workability domain: Considers if and how healthcare workers require extensive training before they can use A&F intervention effectively -Would ideally focus on skill development, competence and ability that A&F intervention requires to affect behaviour in practice. E.g. In Conn *et al* [53], A&F intervention had to address skills and knowledge of the guidelines given that no formal training was provided, consistency with champions’ existing abilities was essential. Sites where high champion turnover was experienced were explained by a lack of coherence with skills. |
| (2) Skills |  |  |
| (3) Social/professional role and identity | Normalisation Process Theory | NPT Coherence's communal specification domain: Considers how A&F intervention promote a shared sense of its purpose in target healthcare givers. Focuses on how A&F interventions highlight a coherent set of behaviours and displayed personal qualities of an individual at the work setting. E.g. Gunn *et al* [54] study -which had an audit and feedback component – targeting the doubts practice nurses had regarding their role in delivering depression care |
| (4) Beliefs about capabilities | Theory of Planned Behaviour | TPB’s Perceived Behavioural Control: Focuses on an individual's perception of the ease or difficulty of performing the behaviour of interest. E.g. Hutchinson *et al* [55] A&F study explored perceived behavioural control when dealing with the perception of the ease or difficulty of performing the medication error reporting behaviour, given that the intent to report errors may be influenced by other factors that ultimately lead to non-reporting behaviour. |
| (5) Beliefs about consequences | Normalisation Process Theory  Theory of Planned Behaviour | NPT Coherence: What benefit does A&F interventions bring and to whom?  Are these benefits likely to be valued by potential participants? - Focuses on consequents on an outcome of behaviour given A&F adoption while also giving credence to rewards (proximal/distal, valued /not valued, probable /improbable). E.g. In Conn *et al.* [53] multi-component study to enhanced recovery after surgery programme, A&F intervention had to address health workers’ concern that a specific intervention negatively affected the patients post operation.  TPB Attitudes - In settings where individuals have strong opinions and attitudes, e.g. Hutchinson *et al* [55] sought to address attitudes dictating reporting of critical incidents (medication error) due to a perception that if no harm is caused, the errors were not worthy of reporting. |
| (6) Motivation and goals | Control Theory  Theory of Planned Behaviour | Control theory: Intentions are translated into action - Focuses on goal / target setting process that establishes specific time based behaviour targets that are measurable, achievable and realistic e.g. Gude *et al*. [35] use of web based A&F intervention to help multidisciplinary teams translate intentions to goals with action plans.  TPB Behavioural intention - Targets motivational factors that influence a given behaviour where the stronger the intention to perform the behaviour, the more likely the behaviour will be performed. Within A&F it is directed at the nature of the behaviour including type, frequency, duration, and intensity, whether routine or habitual, of current behaviour and its intensity when coded in motivation & goals. e.g. Hutchinson *et al* [55] in their A&F intervention, posit that inclusion of TPB- thought to influence both behaviour and intent - might help expand the understanding of the context and constraints on behaviour given that intentions do not always predict behaviour. |
| (7) Memory, attention and decision process | Normalisation Process Theory | NPT Reflexive monitoring: Considers how are health workers likely to perceive A&F intervention once it has been in use for a while, focusing selectively on A&F aspects perceived as advantageous for patients or staff. For example, how relevant cues with A&F might influence the cognitive process of choosing between two or more alternatives of quality improvement strategies e.g. Conn *et al* [53] A&F component helped address health workers’ challenges of continually readily available performance information to show the impact of what they’re doing and help cognitive limitations such as forgetting. |
| (8) Environmental context and resources | Normalisation Process Theory  Control Theory | NPT Collective action: Consider the impact A&F intervention will it have on division of labour, resources, power, and responsibility between different professional groups - Focus is given on any circumstance of a person’s situation or environment that discourages or encourages the development of skills and abilities, independence, social competence, and adaptive behaviour. E.g. Conn *et al* [53] reported that from anaesthesia perspective, easy to implement partly because the guidelines fell into the routine context of work anyway and with available resources including manpower.  Control Theory’s External Disturbances – in Gude *et al* [35] reported presence of environmental stressors in the rollout of electronic patient record systems whose rollout was posited to have conflicted with the time and resources available for working on actual performance improvement. Additionally, due to clinicians disagreeing with the benchmarks, deeming improvement unfeasible, and not considering some indicators as essential aspect of care quality, this impeded intention to improve practice and possibly nullified A&F effects. |
| (9) Social influences | Theory of Planned Behaviour | TPB Subjective norms - This refers to the belief about whether most people approve/disapprove of the behaviour. It relates to a person's beliefs about whether peers or people of importance to the person think he/she should engage in the behaviour e.g. social pressure in a clinical team that every health worker perform key tasks e.g. Hutchinson *et al* [55] within an A&F intervention to improve medication error reporting, propose to implement TPB in-part because it addresses "...the perceived social pressure to comply with a behaviour..." |
| (11) Behaviour regulation | Control Theory | Control theory: How action impacts the outcome of interest - Focuses on the action or process of forming a plan regarding an outcome to be improved when tied to reflection on the ability to modify one's behaviour in response to situation. Gude *et al.* [35] use of web based A&F intervention used Plan-Do-Study-Act cycles informed by Control Theory to support routine evaluation of performance practice and development of improvement strategy. |
| (12) Nature of the behaviours | Normalisation Process Theory | NPT’s ‘normalization’ aspect represents a process of creating habit, and therefore as a theory, captures some of constructs within the ‘nature of the behaviour’ domain. E.g. in Conn *et al* [53], NPT was used in organizing a change model that elucidates the individual and collective cognitive and social processes at work in the intervention implementation to encourage sustainable change in surgical practice and patient care broadly and systemise it. |
| *Note: Normalisation Process Theory (NPT)* [24]*. Theory of Planned Behaviour (TPB)* [26]*. Control Theory* [27]*. NPT mapping to TDF was tentative given that It was not*  *specifically included in the work that informed the development of TDF.* | | |
