# Supplementary material for: A systematic review of electronic audit and feedback: intervention effectiveness and use of behaviour change theory
Source: Implement Sci. 2017 May 12;12:61. doi: 10.1186/s13012-017-0590-z (PMC5427645; doi:10.1186/s13012-017-0590-z)
Supplement: Supplementary file 2 — Search strategy. (DOCX 62 kb) [file 13012_2017_590_MOESM2_ESM.docx]

## **S2: Search Strategy**

### MEDLINE, Ovid

| [# ▲](http://ovidsp.uk.ovid.com/sp-3.18.0b/ovidweb.cgi?&S=LAJPPDBLGIHFIFONFNJKHGPFHLIPAA00&Sort+Sets=descending) | **Searches** | **Results** |
| --- | --- | --- |
| 1 | (audit* adj3 feedback).tw. | 1951 |
| 2 | Clinical Audit/ | 1117 |
| 3 | Medical Audit/ | 15838 |
| 4 | Nursing Audit/ | 3014 |
| 5 | Dental Audit/ | 410 |
| 6 | Management Audit/ | 2464 |
| 7 | Benchmarking/ | 11335 |
| 8 | "Commission on Professional and Hospital Activities"/ | 239 |
| 9 | Feedback/ | 27501 |
| 10 | Feedback, Psychological/ | 2820 |
| 11 | Utilization Review/ | 7572 |
| 12 | Drug Utilization Review/ | 3328 |
| 13 | Concurrent Review/ | 378 |
| 14 | Peer Review, Health Care/ | 1367 |
| 15 | (audit or audits or auditing).tw. | 27697 |
| 16 | feedback.tw. | 86910 |
| 17 | (review adj3 record?).tw. | 11502 |
| 18 | chart review.tw. | 24132 |
| 19 | (practice data or hospital* data).tw. | 3825 |
| 20 | benchmark*.tw. | 19005 |
| 21 | or/2-20 | 211110 |
| 22 | exp Health Personnel/ | 427113 |
| 23 | exp Hospitals/ | 237807 |
| 24 | exp Professional Practice/ | 232166 |
| 25 | Family Practice/ | 63036 |
| 26 | Professional Competence/ | 21977 |
| 27 | Clinical Competence/ | 74956 |
| 28 | Physician's Practice Patterns/ | 47931 |
| 29 | Nurse's Practice Patterns/ | 1567 |
| 30 | Dentist's Practice Patterns/ | 1863 |
| 31 | Quality Assurance, Health Care/ | 52512 |
| 32 | Quality of Health Care/ | 62864 |
| 33 | ((health* personnel or health care personnel or physician? or doctor? or clinician? or nurse? or provider? or practitioner? or resident? or professional? or nursing or clinical) adj3 (skill or skills or behaviour or behavior or competence)).tw. | 30991 |
| 34 | ((clinical or medical or dental or private or general or family or professional or hospital?) adj practice?).tw. | 186620 |
| 35 | (practice pattern? or pattern of practice).tw. | 5358 |
| 36 | (quality adj (assurance or improvement or control)).tw. | 65547 |
| 37 | (health care quality or healthcare quality or quality of healthcare or quality of health care or quality of care).tw. | 39256 |
| 38 | performance.tw. | 561200 |
| 39 | ((influenc* or chang*) adj3 (behaviour* or behavior*)).tw. | 54654 |
| 40 | or/22-39 | 1784319 |
| 41 | User-Computer Interface/ | 31954 |
| 42 | Web Browser/ | 264 |
| 43 | Decision Making, Computer-Assisted/ | 2571 |
| 44 | Software Design/ | 5426 |
| 45 | (user interface or computer interface or software interface or graphical interface or digital interface).tw. | 5766 |
| 46 | Computers/ or Computer Systems/ | 61530 |
| 47 | Software/ or Software Validation/ | 90950 |
| 48 | (usability or interaction design).tw. | 5628 |
| 49 | or/41-48 | 178218 |
| 50 | randomized controlled trial.pt. | 428488 |
| 51 | controlled clinical trial.pt. | 91564 |
| 52 | (randomi* or randomly).tw. | 609924 |
| 53 | or/50-52 | 814853 |
| 54 | 21 and 40 and 49 | 2741 |
| 55 | ((online or web-based or computer* or electronic or digital or software or dashboard* or panel view* or panel support) adj3 (audit* or feedback)).tw. | 1377 |
| 56 | 54 or 55 | 4000 |
| 57 | 56 and 53 | 485 |
| 58 | limit 57 to (abstracts and english language and humans) | 454 |

### EMBASE, Ovid

| [# ▲](http://ovidsp.uk.ovid.com/sp-3.18.0b/ovidweb.cgi?&S=LAJPPDBLGIHFIFONFNJKHGPFHLIPAA00&Sort+Sets=descending) | **Searches** | **Results** |
| --- | --- | --- |
| 1 | (audit* adj3 feedback).tw. | 2736 |
| 2 | Medical Audit/ | 46391 |
| 3 | Feedback System/ | 72606 |
| 4 | Negative Feedback/ | 13287 |
| 5 | Positive Feedback/ | 7793 |
| 6 | "Utilization Review"/ | 64520 |
| 7 | "Medical Record Review"/ | 78978 |
| 8 | (audit or audits or auditing).tw. | 56926 |
| 9 | feedback.tw. | 123052 |
| 10 | (review adj3 record?).tw. | 18772 |
| 11 | chart review.tw. | 49508 |
| 12 | (practice data or hospital* data).tw. | 6896 |
| 13 | benchmark*.tw. | 28718 |
| 14 | or/2-13 | 420973 |
| 15 | exp Health Care Personnel/ | 1144336 |
| 16 | exp Hospital/ | 890179 |
| 17 | exp Professional Practice/ | 304003 |
| 18 | Professional Competence/ | 27563 |
| 19 | Nursing Competence/ | 681 |
| 20 | Clinical Competence/ | 48917 |
| 21 | Health Care Quality/ | 208334 |
| 22 | Quality Control/ | 143471 |
| 23 | ((health* personnel or health care personnel or physician? or doctor? or clinician? or nurse? or provider? or practitioner? or resident? or professional? or nursing or clinical) adj3 (skill or skills or behaviour or behavior or competence)).tw. | 42770 |
| 24 | ((clinical or medical or dental or private or general or family or professional or hospital?) adj practice?).tw. | 275647 |
| 25 | (practice pattern? or pattern of practice).tw. | 8559 |
| 26 | (quality adj (assurance or improvement or control)).tw. | 104523 |
| 27 | (health care quality or healthcare quality or quality of healthcare or quality of health care or quality of care).tw. | 56215 |
| 28 | performance.tw. | 797192 |
| 29 | ((influenc* or chang*) adj3 (behaviour* or behavior*)).tw. | 75383 |
| 30 | or/15-29 | 3260609 |
| 31 | User-Computer Interface/ | 18760 |
| 32 | Web Browser/ | 4543 |
| 33 | Decision Making, Computer-Assisted/ | 15015 |
| 34 | Software Design/ | 180581 |
| 35 | (user interface or computer interface or software interface or graphical interface or digital interface).tw. | 8081 |
| 36 | Computers/ or Computer Systems/ | 98518 |
| 37 | Software/ or Software Validation/ | 185723 |
| 38 | (usability or interaction design).tw. | 8874 |
| 39 | or/31-38 | 307121 |
| 40 | Randomized Controlled Trial/ | 417141 |
| 41 | (randomi* or randomly).tw. | 911799 |
| 42 | 40 or 41 | 1010684 |
| 43 | 14 and 30 and 39 | 7820 |
| 44 | ((online or web-based or computer* or electronic or digital or software or dashboard* or panel view* or panel support) adj3 (audit* or feedback)).tw. | 2239 |
| 45 | 30 and 44 | 1120 |
| 46 | 43 or 45 | 8621 |
| 47 | 42 and 46 | 740 |
| 48 | limit 47 to (abstracts and human and english language and exclude medline journals) | 30 |

### CINAHL Plus Through EBSCOhost

| **#** | **Query** | **Results** |
| --- | --- | --- |
| S54 | S36 AND S45 AND S51 AND S53 | 51 |
| S53 | S13 or S52 | 62,774 |
| S52 | TI ( audit* and feedback ) or AB ( audit* and feedback ) | 1,100 |
| S51 | S46 OR S47 OR S48 OR S49 OR S50 | 283,212 |
| S50 | TI ( ( randomi* or randomly ) ) or AB( ( randomi* or randomly ) ) | 157,662 |
| S49 | (MH “Simple Random Sample”) | 417 |
| S48 | (MH ”Random Sample”) | 26,984 |
| S47 | (MH “Random Assignment”) | 41,483 |
| S46 | (MH “Clinical Trials”) | 124,064 |
| S45 | S37 OR S38 OR S39 OR S40 OR S41 OR S42 OR S43 OR S44 | 32,915 |
| S44 | TI ("usability" or "interaction design") or AB ("usability" or "interaction design") | 2,511 |
| S43 | (MH "Software") | 21,117 |
| S42 | (MH "Computer Systems") | 1,568 |
| S41 | TI ("user interface" or "computer interface" or "software interface" or "graphical interface" or "digital interface") or AB ("user interface" or "computer interface" or "software interface" or "graphical interface" or "digital interface") | 736 |
| S40 | (MH "Software Design") | 2,196 |
| S39 | (MH "Decision Making, Computer-Assisted") | 59 |
| S38 | (MH "Web Browsers") | 304 |
| S37 | (MH "User-Computer Interface") | 7,614 |
| S36 | S14 or S15 or S16 or S17 or S18 or S19 or S20 or S21 or S22 or S23 or S24 or S25 or S26 or S27 or S28 or S29 or S30 or S31 or S32 or S33 or S34 or S35 | 854,395 |
| S35 | TI ( influenc* N3 behaviour* or influenc* N3 behavior* or chang* N3 behaviour* or chang* N3 behavior* ) or AB ( influenc* N3 behaviour* or influenc* N3 behavior* or chang* N3 behaviour* or chang* N3 behavior* ) | 16,528 |
| S34 | TI performance or AB performance | 97,875 |
| S33 | TI ( “health care quality” or “healthcare quality” or quality W1 healthcare or quality W2 care ) or AB ( “health care quality” or “healthcare quality” or quality W1 healthcare or quality W2 care ) | 26,309 |
| S32 | TI ( quality W0 assurance or quality W0 improvement or quality W0 control ) or AB ( quality W0 assurance or quality W0 improvement or quality W0 control ) | 17,053 |
| S31 | TI practice N1 pattern* or AB practice N1 pattern* | 2,130 |
| S30 | TI ( clinical W0 practice* or medical W0 practice* or dental W0 practice* or private W0 practice* or general W0 practice* or family W0 practice* or professional W0 practice* or hospital* W0 practice* ) or AB ( clinical W0 practice* or medical W0 practice* or dental W0 practice* or private W0 practice* or general W0practice* or family W0practice* or professional W0 practice* or hospital* W0 practice* ) | 56,391 |
| S29 | TI ( “health personnel” N3 competence or “healthcare personnel” N3 competence or “health care personnel” N3 competence or physician N3 competence or physicians N3 competence or doctor N3 competence or doctors N3 competence or clinician N3 competence or clinicians N3 competence or nurse N3 competence or nurses N3 competence or provider N3 competence or providers N3 competence or practitioner N3 competence or practitioners N3 competence or resident N3 competence or residents N3 competence or professional N3 competence or professionals N3 competence or nursing N3 competence or clinical N3 competence ) or AB ( “health personnel”N3 competence or “healthcare personnel”N3 competence or “health care personnel” N3 competence or physician N3 competence or physicians N3 competence or doctor N3 competence or doctors N3 competence or clinician N3 competence or clinicians N3 competence or nurse N3 competence or nurses N3 competence or provider N3 competence or providers N3 competence or practitioner N3 competence or practitioners N3 competence or resident N3 competence or residents N3 competence or professional N3 competence or professionals N3 competence or nursing N3 competence or clinical N3 competence ) | 3,777 |
| S28 | TI ( “health personnel” N3 behavior or “healthcare personnel” N3 behavior or “health care personnel” N3 behavior or physician N3 behavior or physicians N3 behavior or doctor N3 behavior or doctors N3 behavior or clinician N3 behavior or clinicians N3 behavior or nurse N3 behavior or nurses N3 behavior or provider N3 behavior or providers N3 behavior or practitioner N3 behavior or practitioners N3 behavior or resident N3 behavior or residents N3 behavior or professional N3 behavior or professionals N3 behavior or nursing N3 behavior or clinicalN3 behavior ) or AB ( “health personnel”N3 behavior or “healthcare personnel” N3 behavior or “health care personnel” N3 behavior or physician N3 behavior or physicians N3 behavior or doctor N3 behavior or doctors N3 behavior or clinician N3 behavior or clinicians N3 behavior or nurse N3 behavior or nurses N3 behavior or provider N3 behavior or providers N3 behavior or practitioner N3 behavior or practitioners N3 behavior or resident N3 behavior or residents N3 behavior or professional N3 behavior or professionals N3 behavior or nursing N3 behavior or clinical N3 behavior ) | 6,741 |
| S27 | TI ( “health personnel” N3 behaviour or “healthcare personnel” N3 behaviour or “health care personnel” N3 behaviour or physician N3 behaviour or physicians N3 behaviour or doctor N3 behaviour or doctors N3 behaviour or clinician N3 behaviour or clinicians N3 behaviour or nurse N3 behaviour or nurses N3 behaviour or provider N3 behaviour or providers N3 behaviour or practitioner N3 behaviour or practitioners N3 behaviour or resident N3 behaviour or residents N3 behaviour or professional N3 behaviour or professionals N3 behaviour or nursing N3 behaviour or clinical N3 behaviour ) or AB ( “health personnel” N3 behaviour or “healthcare personnel” N3 behaviour or “health care personnel” N3 behaviour or physician N3 behaviour or physicians N3 behaviour or doctor N3 behaviour or doctors N3 behaviour or clinician N3 behaviour or clinicians N3 behaviour or nurse N3 behaviour or nurses N3 behaviour or provider N3 behaviour or providers N3 behaviour or practitioner N3 behaviour or practitioners N3 behaviour or resident N3 behaviour or residents N3 behaviour or professional N3 behaviour or professionals N3 behaviour or nursing N3 behaviour or clinical N3 behaviour ) | 6,518 |
| S26 | TI ( “health personnel”N3 skills or “healthcare personnel”N3 skills or “health care personnel”N3 skills or physicianN3 skills or physicians N3 skills or doctor N3 skills or doctors N3 skills or clinician N3 skills or clinicians N3 skills or nurse N3 skills or nurses N3 skills or provider N3 skills or providers N3 skills or practitioner N3 skills or practitioners N3 skills or resident N3 skills or residents N3 skills or professional N3 skills or professionals N3 skills or nursing N3 skills or clinical N3 skills ) or AB ( “health personnel”N3 skills or “healthcare personnel” N3 skills or “health care personnel” N3 skills or physician N3 skills or physicians N3 skills or doctor N3 skills or doctors N3 skills or clinician N3 skills or clinicians N3 skills or nurse N3 skills or nurses N3 skills or provider N3 skills or providers N3 skills or practitioner N3 skills or practitioners N3 skills or resident N3 skills or residents N3 skills or professional N3 skills or professionals N3 skills or nursing N3 skills or clinical N3 skills ) | 11,766 |
| S25 | TI ( “health personnel” N3 skill or “healthcare personnel” N3 skill or “health care personnel” N3 skill or physician N3 skill or physicians N3 skill or doctor N3 skill or doctors N3 skill or clinician N3 skill or clinicians N3 skill or nurse N3 skill or nurses N3 skill or provider N3 skill or providers N3 skill or practitioner N3 skill or practitioners N3 skill or resident N3 skill or residents N3 skill or professional N3 skill or professionals N3 skill or nursing N3 skill or clinical N3 skill ) or AB ( “health personnel” N3 skill or “healthcare personnel” N3 skill or “health care personnel” N3 skill or physician N3 skill or physicians N3 skill or doctor N3 skill or doctors N3 skill or clinician N3 skill or clinicians N3 skill or nurse N3 skill or nurses N3 skill or provider N3 skill or providers N3 skill or practitioner N3 skill or practitioners N3 skill or resident N3 skill or residents N3 skill or professional N3 skill or professionals N3 skill or nursing N3 skill or clinical N3 skill ) | 9,774 |
| S24 | (MH “Quality of Nursing Care”) | 9,503 |
| S23 | (MH “Quality of Health Care”) | 51,587 |
| S22 | (MH “Quality Assurance”) | 17,377 |
| S21 | (MH “Prescribing Patterns”) | 3,675 |
| S20 | (MH “Practice Patterns”) | 11,157 |
| S19 | (MH “Nursing Skills”) | 3,183 |
| S18 | (MH “Clinical Competence”) | 28,201 |
| S17 | (MH “Professional Competence”) | 11,628 |
| S16 | (MH “Professional Practice+”) | 208,254 |
| S15 | (MH “Hospitals+”) | 84,869 |
| S14 | (MH “Health Personnel+”) | 405,648 |
| S13 | S1 or S2 or S3 or S4 or S5 or S6 or S7 or S8 or S9 or S10 or S11 or S12 | 62,774 |
| S12 | TI benchmark* or AB benchmark* | 5,144 |
| S11 | TI hospital* W0 data or AB hospital* W0 data | 1,136 |
| S10 | TI "practice data" or AB "practice data" | 424 |
| S9 | TI “chart review” or AB “chart review” | 6,358 |
| S8 | TI review N3 record* or AB review N3 record* | 4,235 |
| S7 | TI feedback or AB feedback | 16,668 |
| S6 | TI ( audit or audits or auditing or feedback ) or AB ( audit or audits or auditing or feedback ) | 29,399 |
| S5 | (MH "Utilization Review") | 1,758 |
| S4 | (MH “Feedback”) | 8,443 |
| S3 | (MH “Benchmarking”) | 5,606 |
| S2 | (MH “Nursing Audit”) | 874 |
| S1 | (MH “Audit”) | 12,947 |

### CENTRAL (Including EPOC)

| **ID** | **Search** | **Hits** |
| --- | --- | --- |
| #1 | MeSH descriptor: [Clinical Audit] this term only | 14 |
| #2 | MeSH descriptor: [Medical Audit] this term only | 270 |
| #3 | MeSH descriptor: [Nursing Audit] this term only | 49 |
| #4 | MeSH descriptor: [Dental Audit] this term only | 4 |
| #5 | MeSH descriptor: [Management Audit] this term only | 4 |
| #6 | MeSH descriptor: [Benchmarking] this term only | 112 |
| #7 | MeSH descriptor: [Commission on Professional and Hospital Activities] this term only | 4 |
| #8 | MeSH descriptor: [Feedback] this term only | 1093 |
| #9 | MeSH descriptor: [Feedback, Psychological] this term only | 401 |
| #10 | MeSH descriptor: [Utilization Review] this term only | 138 |
| #11 | MeSH descriptor: [Drug Utilization Review] this term only | 133 |
| #12 | MeSH descriptor: [Concurrent Review] this term only | 3 |
| #13 | MeSH descriptor: [Peer Review, Health Care] this term only | 40 |
| #14 | (audit or audits or auditing or feedback or benchmark*):ti,ab | 8092 |
| #15 | (audit* near/3 feedback):ti,ab | 335 |
| #16 | (review near/3 record* or chart next review or practice next data or hospital* next data):ti,ab | 1118 |
| #17 | (#1 or #2 or #3 or #4 or #5 or #6 or #7 or #8 or #9 or #10 or #11 or #12 or #13 or #14 or #15 or #16) | 9979 |
| #18 | MeSH descriptor: [Health Personnel] explode all trees | 7042 |
| #19 | MeSH descriptor: [Hospitals] explode all trees | 3458 |
| #20 | MeSH descriptor: [Professional Practice] explode all trees | 3808 |
| #21 | MeSH descriptor: [Family Practice] this term only | 2174 |
| #22 | MeSH descriptor: [Professional Competence] this term only | 238 |
| #23 | MeSH descriptor: [Clinical Competence] this term only | 2450 |
| #24 | MeSH descriptor: [Physician's Practice Patterns] this term only | 1240 |
| #25 | MeSH descriptor: [Nurse's Practice Patterns] this term only | 92 |
| #26 | MeSH descriptor: [Dentist's Practice Patterns] this term only | 19 |
| #27 | MeSH descriptor: [Quality Assurance, Health Care] this term only | 787 |
| #28 | MeSH descriptor: [Quality of Health Care] this term only | 1036 |
| #29 | MeSH descriptor: [Outcome and Process Assessment (Health Care)] this term only | 2168 |
| #30 | (health*NEXT personnel or "health care personnel" or physician* or doctor* or clinician* or nurse* or provider* or practitioner* or resident* or professional* or nursing or clinical) near/3 (skill or skills or behaviour or behavior or competence):ti,ab | 1859 |
| #31 | (clinical or medical or dental or private or general or family or professional or hospital*) next practice*:ti,ab | 15304 |
| #32 | (practice near/2 pattern*):ti,ab | 263 |
| #33 | quality next (assurance or improvement or control):ti,ab | 2008 |
| #34 | (health* or care) near/2 quality:ti,ab | 9054 |
| #35 | performance:ti,ab | 42897 |
| #36 | (influenc* near/3 behaviour* or influenc* near/3 behavior* or chang* near/3 behaviour* or chang* near/3 behavior*):ti,ab | 5607 |
| #37 | (#18 or #19 or #20 or #21 or #22 or #23 or #24 or #25 or #26 or #27 or #28 or #29 or #30 or #31 or #32 or #33 or #34 or #35 or #36) | 87066 |
| #38 | (#17 and #37) | 3818 |
| #39 | MeSH descriptor: [User-Computer Interface] explode all trees | 1132 |
| #40 | MeSH descriptor: [Web Browser] explode all trees | 3 |
| #41 | MeSH descriptor: [Decision Making, Computer-Assisted] explode all trees | 4481 |
| #42 | MeSH descriptor: [Software Design] explode all trees | 76 |
| #43 | (user interface or computer interface or software interface or graphical interface or digital interface):ti,ab | 297 |
| #44 | MeSH descriptor: [Computer Systems] explode all trees | 4022 |
| #45 | MeSH descriptor: [Software] explode all trees | 2451 |
| #46 | (usability or interaction design):ti,ab | 4288 |
| #47 | MeSH descriptor: [Registries] explode all trees | 922 |
| #48 | (#39 or #40 or #41 or #42 or #43 or #44 or #45 or #46 or #47) | 14993 |
| #49 | #38 and #48 in Trials | 375 |
