# Supplementary material for: A systematic review of electronic audit and feedback: intervention effectiveness and use of behaviour change theory
Source: Implement Sci. 2017 May 12;12:61. doi: 10.1186/s13012-017-0590-z (PMC5427645; doi:10.1186/s13012-017-0590-z)
Supplement: Supplementary file 3 — TDF domains and constructs. (DOCX 55 kb) [file 13012_2017_590_MOESM3_ESM.docx]

## **S3: TDF domains and constructs**

| **Theoretical Domains Framework, Domains and Constructs Coding manual (Adapted from Michie et al 2005 and Cane et al 2012) [45]** | |
| --- | --- |
| Domains and Constructs | Examples of relevant data |
| **1: Knowledge** *(An awareness of the existence of something)*   - Knowledge *(including knowledge of condition /scientific rationale)* - Procedural knowledge *(Knowing how to do something)* - Schemas + mindsets + illness representations | - Statements about having/not having/wanting factual or procedural knowledge of when and how to do the behaviour - Statements about having/not having/wanting an understanding of the rationale behind performing the behaviour - I know/do not know/want to know how/when to do the behaviour - I know/do not know/want to know why I should do the behaviour   In the context of this study, knowledge of the condition/scientific rationale could relate to knowledge of obesity determinants, risks in pregnancy, weight management theory etc. Knowledge of these factors may be both correct and incorrect knowledge. |
| **2: Skills** *(An ability or proficiency acquired through practice)*   - Skills development *(The gradual acquisition or advancement through progressive stages of an ability or proficiency acquired through training and practice)* - Competence *(One's repertoire of skills, and ability especially as it is applied to a task or set of tasks)* - Ability *(Competence or capacity to perform a physical or mental act. Ability may be either unlearned or acquired by education and practice* - Interpersonal skills *(An aptitude enabling a person to carry on effective relationships with others, such as an ability to cooperate, to assume appropriate social responsibilities or to exhibit adequate flexibility)* - Practice *(Repetition of an act, behaviour, or series of activities, often to improve performance or acquire a skill)* - Skill assessment *(A judgment of the quality, worth, importance, level, or value of an ability or proficiency acquired through training and practice)* - Coping strategies | - Statements describing techniques/capability/skills used/how they do the behaviour in practice - Statements about wanting to develop/improve skills in doing the behaviour - In practice, I do x,y,z to help me perform the behaviour - I would like training in how best to do the behaviour/we haven’t had any training in how to do the behaviour   In the context of this study, skills may be interpersonal skills (e.g. using empathy, practice, encouragement, sensitivity, practical advice, promote benefits, non-judgemental approach, terminology, normalisation, communication skills etc). May also involve skills adopted to cope in the absence of specific skills training related to behaviours (e.g. ‘We’ve never been told how to broach categorising women as obese so I try to normalise it by x, y and z’) |
| **3: Social or Professional Role and Identity (Self-standards)** *(A coherent set of behaviours and displayed personal qualities of an individual in a social or work setting)*   - Professional identity *(The characteristics by which an individual is recognised relating to, connected with or befitting a particular profession)* - Professional role *(The behaviour considered appropriate for a particular kind of work or social position)* - Social identity *(The set of behavioural or personal characteristics by which an individual is recognizable [and portrays] as a member of a social group)* - Identity *(An individual's sense of self defined by a) a set of physical and psychological characteristics that is not wholly shared with any other person and b) a range of social and interpersonal affiliations (e.g., ethnicity) and social roles)* - Professional boundaries *(The bounds or limits relating to, or connected with a particular profession or calling)* - Group identity *(The set of behavioural or personal characteristics by which an individual is recognizable [and portrays] as a member of a group)* - Organisational commitment *(An employee's dedication to an organisation and wish to remain part of it. Organisational commitment is often described as having both an emotional or moral element and a more prudent element)* - Social and group norms - Alienation (Estrangement from one’s social group; a deep-seated sense of dissatisfaction with one’s personal experiences that can be a source of lack of trust in one’s social or physical environment or in oneself; the experience of separation between thoughts and feelings) | - Statements relating to how healthcare professionals see themselves - Statements relating to the extent they view the behaviour as a characteristic/ feature/ meaningful aspect/ representative of their professional role - Statements relating to the extent their personal identity influences doing the behaviour - It is/isn’t part of our role/job/profession/responsibility to do the behaviour - My [personal identity] impacts on how/whether I perform the behaviour   In the context of this study, professional role may relate to the extent that healthcare professionals feel that providing obesity and weight management support is part of their professional role, and the roles of other healthcare professional groups. Personal identity may relate to healthcare professionals gender or own weight status and the impact this has on providing obesity and weight management support. |
| **4: Beliefs about Capabilities (Self-efficacy)** *(Acceptance of the truth, reality, or validity about an ability, talent, or facility that a person can put to constructive use)*   - Self-confidence *(Self-assurance or trust in one's own abilities, capabilities and judgment)* - Perceived competence *(An individual's belief in his or her ability to learn and execute skills)* - Self-efficacy *(An individual's capacity to act effectively to bring about desired results, as perceived by the individual)* - Perceived behavioural control *(An individual's perception of the ease or difficulty of performing the behaviour of interest)* - Self-esteem *(The degree to which the qualities and characteristics contained in one's self- concept are perceived to be positive)* - Empowerment *(The promotion of the skills, knowledge and confidence necessary to take great control of one's life as in certain educational or social schemes; the delegation of increased decision-making powers to individuals or groups in a society or organisation)* - Professional confidence *(An individual's belief in his or her repertoire of skills, and ability especially as it is applied to a task or set of tasks)*  Control of behaviour and material and social environment - Optimism (The confidence that things will happen for the best or that desired goals will be attained) - Pessimism (The attitude that things will go wrong and that people's wishes or aims are unlikely to be fulfilled) | - Evaluative statements of healthcare professionals’ confidence, judgements about their competence and control in their ability (or inability) to perform the behaviour o I do/don’t feel confident/able/capable/competent to do the behaviour o I find it difficult/easy etc to do the behaviour - I feel that I have/don’t have control in doing the behaviour - Statements relating to expectations of carrying out a behaviour due to beliefs of competency in performing the behaviour - I know *behaviour+ will be/won’t be successful because I am/am not very efficient at that task   In the context of this study, beliefs about capabilities relates to healthcare professionals making evaluative judgments on their ability to do the behaviour, for example their confidence in being able to sensitively discuss women’s weight and subsequent risks in pregnancy etc. This would also include expressing optimism/pessimism of effectively discussing obesity, weight management, nutrition etc. based directly upon their believed competence in these behaviours. |
| **5: Beliefs about Consequences (Anticipated outcomes/attitude)** *(Acceptance of the truth, reality, or validity about outcomes of a behaviour in a given situation)*   - Beliefs *(The thing believed; the proposition or set of propositions held true)* - Outcome expectancies *(Cognitive, emotional, behavioural, and affective outcomes that are assumed to be associated with future or intended behaviours. These assumed outcomes can either promote or inhibit future behaviours)* - Characteristics of outcome expectancies *(Characteristics of the cognitive, emotional and behavioural outcomes that individuals believe are associated with future or intended behaviours and that are believed to either promote or inhibit these behaviours. These include whether they are sanctions/rewards, proximal/distal, valued/not valued, probable/improbable, salient/not salient, perceived risks or threats)* - Anticipated regret *(A sense of the potential negative consequences of a decision that influences the choice made: for example, an individual may decide not to make an investment because of the feelings associated with an imagined loss)* - Consequents *(An outcome of behaviour in a given situation)* - Unrealistic optimism *(The inert tendency for humans to over-rate their own abilities and chances of positive outcomes compared to those of other people)* - Salient events / sensitisation / critical Incidents *(Occurrences that one judges to be distinctive, prominent or otherwise significant)* - Attitudes - Contingencies *(A conditional probabilistic relation between two events. Contingencies may be arranged via dependencies or they may emerge by accident)* - Reinforcement *(Increasing the probability of a response by arranging a dependent relationship, or contingency, between the response and a given stimulus. A process in which the frequency of a response is increased by a dependent relationship or contingency with a stimulus)* - Punishment *(The process in which the relationship between a response and some stimulus or circumstance results in the response becoming less probable; a painful, unwanted or undesired event or circumstance imposed as a penalty on a wrongdoer)* - Consequents *(An outcome of behaviour in a given situation)* - Rewards *(proximal / distal, valued / not valued, probable /improbable) (Return or recompense made to, or received by a person contingent on some performance)* - Incentives *(An external stimulus, such as condition or object, that enhances or serves as a motive for behaviour)* - Sanctions *(A punishment or other coercive measure, usually administered by a recognised authority, that is used to penalise and deter inappropriate or unauthorised actions)* | - Statements relating to healthcare professionals’ beliefs/views etc. on the outcome/consequences of doing/not doing the behaviour - Statements can include positive or negative consequences of doing/not doing the behaviour - Statements can include consequences of doing/not doing the behaviour on themselves or their patients - If I do/don’t do the behaviour, x,y,z will happen - Doing the behaviour will have a beneficial/adverse impact on me/my patient - Statements relating to doing the behaviour being directly contingent on receiving rewards or punishments - I do/don’t do x, y, z because otherwise x, y, z will/will not happen - Getting praise/thanked etc. for doing the behaviour encourages me to do it - I do the behaviour because I will be in trouble/get told off/be reported/get reprimanded etc. if I don’t   In the context of this study, beliefs about consequences could relate to healthcare professionals’ beliefs that doing the behaviour will result in negative reactions from women being categorised as obese/damage the midwife-woman relationship etc. Also, willingness to perform the behaviour based on expectations of outcomes (e.g. weight management in pregnancy is pointless/too late to reduce risks etc.). Reinforcement could relate to doing behaviours such as categorising a woman as obese because she needs to have a risk assessment by another department, or if the behaviour is linked with punishments such as litigation/ complaints, or rewards such as continued professional development/personal satisfaction/patient satisfaction rewards etc. |
| **6: Motivation and Goals (Intention)** *(Mental representations of outcomes or end states that an individual wants to achieve)*   - Goals: distal / proximal *(Desired state of affairs of a person or system, these may be closer (proximal) or further away (distal))* - Goal priority *(Order of importance or urgency of end states toward which one is striving)* - Goal / target setting *(A process that establishes specific time based behaviour targets that are measurable, achievable and realistic)* - Goals: autonomous /controlled *(The end state toward which one is striving: the purpose of an activity or endeavour. It can be identified by observing that a person ceases or changes its behaviour upon attaining this state; proficiency in a task to be achieved within a set period of time)* - Intention *(A conscious decision to perform a behaviour or a resolve to act in a certain way)* - Stability of intention/certainty of intention *(Ability of one’s resolve to remain in spite of disturbing influences)* - Transtheoretical model and stages of change *(A five-stage theory to explain changes in people’s health behaviour. It suggests that change takes time, that different interventions are effective at different stages, and that there are multiple outcomes occurring across the stages)* - Intrinsic motivation - Commitment | - Statements relating to the healthcare professionals’ goals/aims/desired end result of doing the behaviour - Statements relating to other goals which may interfere with doing the behaviour - Statements relating to how prioritising goals influences whether or not to do the behaviour - Competing priorities mean I do/don’t do the behaviour - I do/don’t do the behaviour as it will/won’t meet my main goals o I prioritise other behaviours which are more important - Statements relating to healthcare professionals resolve to/the extent they plan to perform the behaviour - I plan to/set out to/aim to /am determined to/want to/don’t want to do the behaviour   In the context of this study, intentions may be a midwife stating how she aims to always discuss diet and nutrition when she sees a pregnant woman or conversely weak intentions may be a lack of intention to discuss diet and nutrition (e.g. I don’t always make a point to discuss it) In the context of this study, healthcare professional’s goals may relate to wanting to support obese pregnant women with their weight-related behaviours to improve their health, their family’s health, pregnancy outcomes, to reduce risks, to make their job easier etc. Goal priorities may relate to the competing topics to cover during antenatal appointments, and how important obesity and weight management is perceived in comparison with other priorities such as smoking cessation etc.  (Intentions – things I want to do; Goals – things I want to achieve) |
| **7: Memory Attention and Decision Processes** *(The ability to retain information, focus selectively on aspects of the environment and choose between two or more alternatives)*   - Memory *(The ability to retain information or a representation of a past experience, based on the mental processes of learning or encoding retention across some interval of time, and retrieval or reactivation of the memory; specific information of a specific past)* - Attention *(A state of awareness in which the senses are focussed selectively on aspects of the environment and the central nervous system is in a state of readiness to respond to stimuli)* - Attention control *(The extent to which a person can concentrate on relevant cues and ignore all irrelevant cues in a given situation)* - Decision making *(The cognitive process of choosing between two or more alternatives, ranging from the relatively clear cut to the complex)* | - Statements relating to time/situations etc. when the healthcare professionals would remember or forget to do the behaviour - Statements relating to relying on cognitive approaches to perform the behaviour/make a quick decision - Statements relating to cognitive limitations such as forgetting/overseeing/not being able to make the decision - We have to discuss so many issues that I forget to do the behaviour - There are so many problems with doing the behaviour that I can’t decide/feel overwhelmed/don’t know where to start - I don’t do the behaviour because I can’t make the decision in the pressure of the situation and competing demands/feel too tired at the end of the day to concentrate to make the right decision   In the context of this study memory, attention and decision processes may relate to the healthcare professionals’ ability to remember to discuss weight/weight management at specific appointments, or due to the complexity of obesity not knowing where to start, or due to having to discuss too many different public health issues they feel overwhelmed and find it difficult to make the decision on how much information or the priority of information to give. |
| **8: Environmental Context and Resources** *(Any circumstance of a person’s situation or environment that discourages or encourages the development of skills and abilities, independence, social competence, and adaptive behaviour)*   - Environmental stressors *(External factors in the environment that cause stress)* - Resources / material resources (availability and management) *(Commodities and human resources used in enacting a behaviour)* - Organisational culture/climate *(A distinctive pattern of thought and behaviour shared by members of the same organisation and reflected in their language, values, attitudes, beliefs and customs)* - Salient events / critical Incidents *(Occurrences that one judges to be distinctive, prominent or otherwise significant)* - Person x environment interaction *(Interplay between the individual and their surroundings)*   Knowledge of task environment *(Knowledge of the social and material context in which a task is undertaken)* | - Describing the presence or absence of tools/resources/ equipment/ services/ organisational structures which facilitate/impede performing the behaviour - Describing how the organisational practice/culture/maternity population facilitates/impedes performing the behaviour - Wanting tools/resources/equipment/services/changes in the organisational structure to facilitate performing the behaviour - We have/don’t have/need services/resources etc. to do the behaviour - The services/resources etc. that we have to do the behaviour are good/ sufficient/ poor/ insufficient etc. - The environment/organisational culture etc. has an impact on doing the behaviour - Examples of the environmental context and resources in this study could be the availability of support services, patient or healthcare professional information, equipment, service-level pathways of care for obesity, time, staffing levels, whether organisation culture prioritises/provides resource for obesity or not etc. (note: in relation to having to prioritise behaviours due to time restrictions then time would be coded as goals) |
| **9: Social Influences** *(Those interpersonal processes that can cause individuals to change their thoughts, feelings, or behaviours)*   - Social pressure *(The exertion of influence on a person or group by another person or group)* - Social norms *(Socially determined consensual standards that indicate a) what behaviours are considered typical in a given context and b) what behaviours are considered proper in the context)* - Group conformity *(The act of consciously maintaining a certain degree of similarity to those in your general social circles)* - Social comparisons *(The process by which people evaluate their attitudes, abilities, or performance relative to others)* - Group norms *(Any behaviour, belief, attitude or emotional reaction held to be correct or acceptable by a given group in society)* - Social support *(The apperception or provision of assistance or comfort to others, typically in order to help them cope with a variety of biological, psychological and social stressors. Support may arise from any interpersonal relationship in an individual’s social network, involving friends, neighbours, religious institutions, colleagues, caregivers or support groups)* - Power/hierarchy *(The capacity to influence others, even when they try to resist this influence)* - Intergroup conflict *(Disagreement or confrontation between two or more groups and their members. This may involve physical violence, interpersonal discord, or psychological tension)* - Group identity *(The set of behavioural or personal characteristics by which an individual is recognizable [and portrays] as a member of a group)* - Learning and modelling *(In developmental psychology the process in which one or more individuals or other entities serve as examples (models) that a child will copy)* - Organisational culture/climate *(A distinctive pattern of thought and behaviour shared by members of the same organisation and reflected in their language, values, attitudes, beliefs and customs)* - Organisational development - Leadership *(The processes involved in leading others, including organising, directing, coordinating and motivating their efforts toward achievement of certain group or organisation goals)* - Team working - Professional boundaries/roles - Management commitment - Supervision - Champions - Social comparisons - Identity *(An individual's sense of self defined by a) a set of physical and psychological characteristics that is not wholly shared with any other person and b) a range of social and interpersonal affiliations (e.g., ethnicity) and social roles)* - Group identity *(The set of behavioural or personal characteristics by which an individual is recognizable [and portrays] as a member of a group)* - Social identity *(The set of behavioural or personal characteristics by which an individual is recognizable [and portrays] as a member of a social group)* - Organisational commitment/alienation - Feedback - Conflict—competing demands, conflicting roles - Change management - Crew resource management   Negotiation | - Statements expressing the influence of others on doing the behaviour (social support, group norms etc.) - I do/don’t do the behaviour this because ‘others’ condone/ support/ advocate/ disapprove/ dictate/ demand it   In the context of this study, others may include individuals or groups of peers, other healthcare professional groups, colleagues, management/authoritative organisations etc. Additionally, when healthcare professionals want patient perspectives, feedback, and experiences to influence their behaviour this would be a social influence (e.g. healthcare professionals want feedback on patient experiences of obesity communication to help develop their communications skills etc.). However, when referring to the interpersonal nature of conducting the behaviour with the patient (e.g. discussing weight management strategies, informing of obesity status) this would be classed as Skills. |
| **10: Emotion** *(A complex reaction pattern, involving experiential, behavioural, and physiological elements, by which the individual attempts to deal with a personally significant matter or event)*   - Fear *(An intense emotion aroused by the detection of imminent threat, involving an immediate alarm reaction that mobilises the organism by triggering a set of physiological changes)* - Anxiety *(A mood state characterised by apprehension and somatic symptoms of tension in which an individual anticipates impending danger, catastrophe or misfortune)* - Affect *(An experience or feeling of emotion, ranging from suffering to elation, from the simplest to the most complex sensations of feelings, and from the most normal to the most pathological emotional reactions)* - Stress *(A state of physiological or psychological response to internal or external stressors)* - Depression *(A mental state that presents with depressed mood, loss of interest or pleasure, feelings of guilt or low self-worth, disturbed sleep or appetite, low energy, and poor concentration)* - Positive / negative affect *(The internal feeling/state that occurs when a goal has/has not been attained, a source of threat has/has not been avoided, or the individual is/is not satisfied with the present state of affairs)* - Burn-out *(Physical, emotional or mental exhaustion, especially in one's job or career, accompanied by decreased motivation, lowered performance and negative attitudes towards oneself and others)* - Cognitive overload / tiredness *(The situation in which the demands placed on a person by mental work are greater than a person’s mental abilities)* - Anticipated regret - Threat | - An expression of their own personal emotional reaction/state to performing the behaviour - Expressing how their emotional reaction/state positively or negatively impacts on them doing the behaviour - I get embarrassed/ upset/ scared/ anxious/ stressed/ depressed/ uncomfortable/ happy/ elated/ relaxed/ pleased etc. when doing the behaviour - I do/don’t want to do the behaviour because it is embarrassing/ upsetting/ elating etc. - I feel sympathy / empathy / sorry for the patient which makes me want to do the behaviour - In the context of this study, emotion relates to the emotional response of the healthcare professional in relation to performing the behaviour, and not the emotional response of the patients to the behaviour (e.g. healthcare professionals being anxious about telling women obesity-related risks rather than women getting upset by risks etc.). Would include emotive response to performing a behaviour irrespective of competence of competence in performing behaviour (e.g. apprehensive of informing a woman she is obese regardless of whether good or bad at broaching the subject) |
| **11: Behavioural Regulation** *(Anything aimed at managing or changing objectively observed or measured actions)*   - Self-monitoring *(A method used in behavioural management in which individuals keep a record of their behaviour, especially in connection with efforts to change or regulate the self; a personality trait reflecting an ability to modify one's behaviour in response to situation)* - Action planning *(The action or process of forming a plan regarding a thing to be done or a deed)* - Barriers and facilitators *(In psychological contexts barriers/facilitators are mental, emotional or behavioural limitations/strengths in individuals or groups)* - Goal / target setting *(A process that establishes specific time based behaviour targets that are measurable, achievable and realistic)* - Implementation intention *(The plan that one creates in advance of when, where and how one will enact a behaviour)* - Goal priority *(Order of importance or urgency of end states toward which one is striving)* - Generating alternatives - Feedback - Moderators of intention-behaviour gap - Project management | - Statements where healthcare professionals want audit/evaluation/feedback on their behaviour - Statements about processes in place/needed to monitor doing the behaviour - Statements about prompts/processes etc. used or required to make the behaviour sustainable/routine/habit - Statements about using conscious effort to ensure the behaviour is carried out - I plan in advance/make notes/use prompts so I don’t forget to do the behaviour.   In the context of this study behavioural regulation may relate primarily to the need for/use of prompts relating to the behaviour such as having specific sections of women’s notes that relate to the behaviours, pathways of care etc. |
| **12: Nature of the behaviours**   - Routine/automatic/habit - Breaking habit *(To discontinue a behaviour or sequence of behaviours that is automatically activated by relevant situational cues)* - Direct experience/past behaviour - Representation of tasks - Stages of change model *(A model that proposes that behaviour change is accomplished through five specific stages: Pre-contemplation, Contemplation, Preparation, Action, and Maintenance)* | Statements referring to –  The nature of the behaviour including type, frequency, (including routines/habits), duration and intensity of past or current behaviour (statements about the future type, frequency, duration and intensity of a behaviour coded in *motivation & goals*).  In the context of this study, this may include the time spent discussing weight management strategies, nutrition, physical activity recommendations in an appointment, reporting the procedure of conducting the behaviour (e.g. we calculate BMI by measuring their weight and height and then use a chart for their BMI status) |

Supplementary Table 3: TDF Constructs used for the coding exercise. All definitions are based on definitions from the American Psychological Associations’ Dictionary of Psychology.
