# Supplementary figures and images for: A systematic review of electronic audit and feedback: intervention effectiveness and use of behaviour change theory
Source: Implement Sci. 2017 May 12;12:61. doi: 10.1186/s13012-017-0590-z (PMC5427645; doi:10.1186/s13012-017-0590-z)

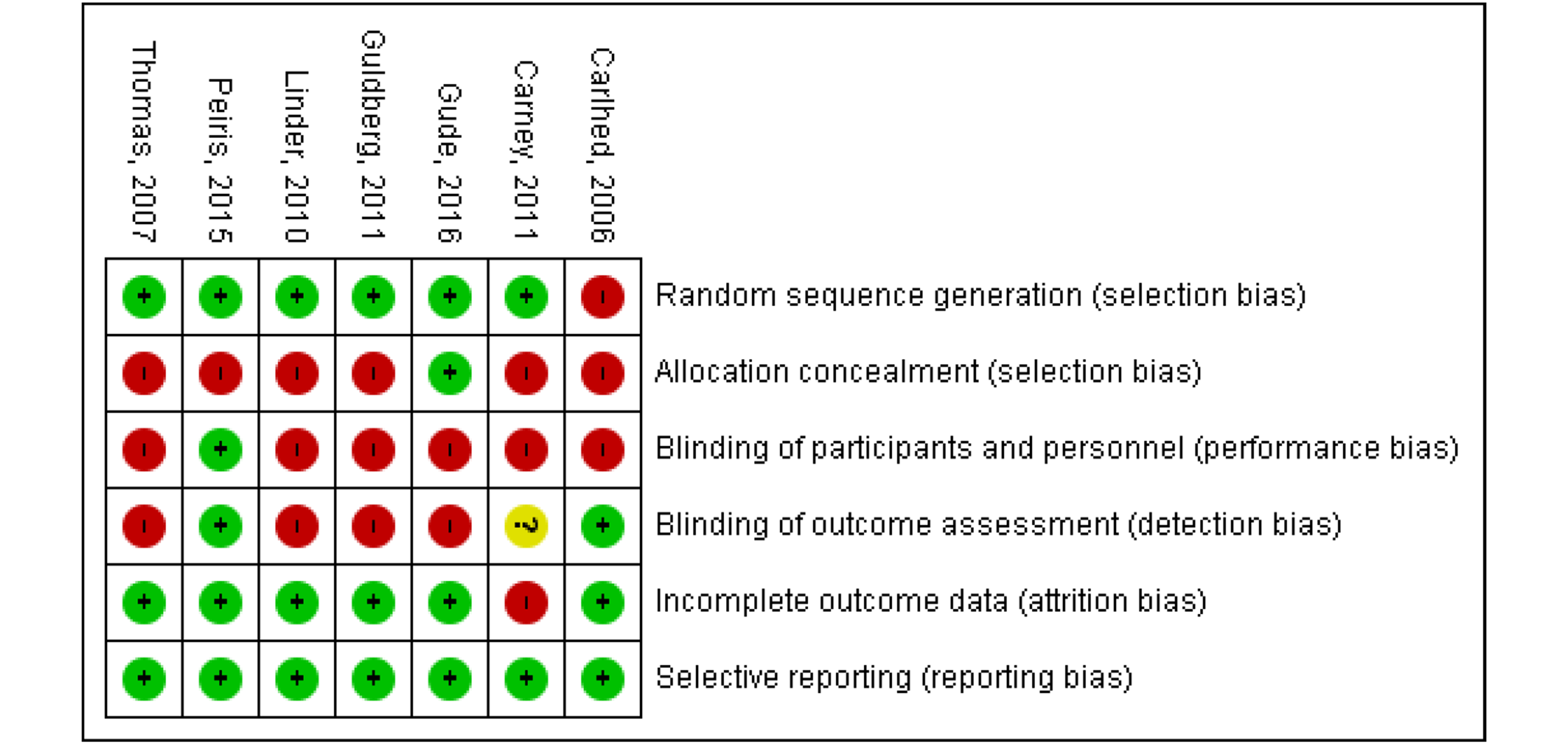

Supplement: Supplementary file 4 — Study specific bias assessment. (TIF 5143 kb) [file 13012_2017_590_MOESM4_ESM.tif]
